# Lyn Delivers Bacteria to Lysosomes for Eradication through TLR2-Initiated Autophagy Related Phagocytosis

**DOI:** 10.1371/journal.ppat.1005363

**Published:** 2016-01-06

**Authors:** Xuefeng Li, Sisi He, Xikun Zhou, Yan Ye, Shirui Tan, Shuang Zhang, Rongpeng Li, Min Yu, Michael C. Jundt, Alec Hidebrand, Yongsheng Wang, Guoping Li, Canhua Huang, Min Wu

**Affiliations:** 1 State Key Laboratory of Biotherapy and Cancer Center, West China Hospital, Sichuan University, and Collaborative Innovation Center for Biotherapy, Chengdu, Sichuan, P.R. China; 2 Department of Biomedical Sciences, University of North Dakota, Grand Forks, North Dakota, United States of America; 3 Department of Thoracic Oncology, West China Hospital, Sichuan University, Chengdu, Sichuan, P.R. China; 4 Inflammations & Allergic Diseases Research Unit, Affiliated Hospital of Luzhou Medical College, Luzhou, Sichuan, P.R. China; University of Michigan Medical School, UNITED STATES

## Abstract

Extracellular bacteria, such as *Pseudomonas aeruginosa* and *Klebsiella pneumoniae*, have been reported to induce autophagy; however, the role and machinery of infection-induced autophagy remain elusive. We show that the pleiotropic Src kinase Lyn mediates phagocytosis and autophagosome maturation in alveolar macrophages (AM), which facilitates eventual bacterial eradication. We report that Lyn is required for bacterial infection-induced recruitment of autophagic components to pathogen-containing phagosomes. When we blocked autophagy with 3-methyladenine (3-MA) or by depleting Lyn, we observed less phagocytosis and subsequent bacterial clearance by AM. Both morphological and biological evidence demonstrated that Lyn delivered bacteria to lysosomes through xenophagy. TLR2 initiated the phagocytic process and activated Lyn following infection. Cytoskeletal trafficking proteins, such as Rab5 and Rab7, critically facilitated early phagosome formation, autophagosome maturation, and eventual autophagy-mediated bacterial degradation. These findings reveal that Lyn, TLR2 and Rab modulate autophagy related phagocytosis and augment bactericidal activity, which may offer insight into novel therapeutic strategies to control lung infection.

## Author Summary

It is vital to establish the mechanistic basis for initiation of host defenses and immune responses that are required to eliminate bacterial infection. This line of inquiry will increase knowledge of bacterial pathogenesis and uncover new insights that can enhance design and effectiveness of novel therapeutics. We demonstrate that TLR-2 is required for inducing Lyn activity in host defense against Pa infection through assistance in autophagosome maturation, and may link autophagy to phagocytosis in a TLR-2-Lyn-dependent manner. Thus, these results may further help to alleviate human acute lung injury/adult respiratory distress syndrome (ALI/ARDS) caused by Gram-negative bacteria.

## Introduction

Gram-negative bacteria, such as *Pseudomonas aeruginosa* (hereafter Pa) and *Klebsiella pneumoniae* (Kp) are formidable threats to human health, imposing huge healthcare costs worldwide. Pa is the fourth most commonly isolated nosocomial pathogen, accounting for 10% of all hospital-acquired infections, while Kp is the third most commonly isolated pathogen from the blood of sepsis patients [1,2]. Increased incidence of antibiotic resistance and lack of effective treatment approaches further limit current interventional options. Alveolar macrophages (AM) are on the front line of host defense with a potential role in eliminating bacterial pathogens by phagocytosis and inflammatory responses [3,4]. Despite decades of extensive research efforts, the role of AM in phagocytosis and clearance of extracellular bacteria remains incompletely understood, which hinders the development of effective therapeutic strategies.

Autophagy is a highly conserved homeostatic mechanism for degrading bulk cellular components during starvation, or other scenarios, to provide the cell with essential nutrients. It has been linked to a wide variety of normal physiological processes including energy metabolism, organelle turnover, growth regulation, and aging [5]. Impaired autophagy can affect the process of various diseases, such as cardiomyopathy, cancer, and infection [6]. Innate immune effectors, such as toll like receptors (TLRs), are important for host defense against pathogens through initiation of phagocytosis and inflammatory response [7]. Autophagy may be modulated following the recognition of conserved pathogen-associated molecular patterns (PAMPs), which interact with host pattern recognition receptors (PRRs), such as TLRs [8,9]. Autophagy can be induced in murine macrophages by several TLR ligands, including poly (I:C) (TLR3), LPS (TLR4) and single strand RNA (TLR7) [7]. Interactions between phagocytes, including AM, and bacteria may critically influence the fate of both pathogens and phagocytes through multiple signaling cascades [10]. However, little is known about whether there is interaction between autophagy and phagocytosis during bacterial invasion.

Further characterization of the mechanistic underpinnings required to launch and execute immune defenses to eliminate bacterial infection is expected to significantly improve our knowledge of bacterial pathogenesis, thereby providing insight into the design of novel and effective therapeutics. One of the central themes in effective host defense is to understand how host cells counteract invasive bacteria, especially participating in the transport of bacteria to lysosomal killing environments for proteolytic digestion. A recent study of the intracellular bacterium *Mycobacterium* showed that the autophagy adaptor SQSTM1 (p62) can enhance delivery of bacterial cytosolic components and increase bacterial killing following phagocytosis [11]. Autophagy adaptors, such as SQSTM1, NDP52 and optineurin, were shown to mediate LC3 recruitment to the ubiquitinated substrate during ubiquitin-dependent xenophagy. Formation of the isolation membrane takes place in the proximity of the early phagosomes. Subsequently, the autophagosome engulfs the pathogen-containing phagosome. In contrast to the double-membraned autophagosome, which is not formed in LC3-associated phagocytosis (LAP), the phagosomal membrane is impacted directly by LC3 [12,13].

Prior studies implicated that the Src kinase Lyn initiates FcγR-mediated phagocytosis and participates in the process of post-phagosome formation by interacting with cytoskeletal proteins [14,15]. In the case of the extracellular bacterium Pa, we discovered that Lyn, lipid rafts, and TLR2 may play a role in phagocytosis [16,17]. Here, we demonstrate that TLR-2 is required for inducing Lyn activity in host defense against Pa infection by facilitating autophagosome maturation. We hypothesized that Lyn-mediated phagocytosis may link autophagy to phagocytosis in a TLR2-Lyn dependent manner. We report that Lyn is a critical upstream signaling component, which expands the concept of general xenophagy [12,18]. In addition, we dissected the molecular and cellular bases regarding how Lyn and autophagy contribute to innate immunity through the eventual degradation of bacterial components.

## Results

### Lyn deficiency decreases phagocytosis and autophagy against Pa infection

To analyze the expression pattern of autophagy-related genes, we determined their mRNAs in mouse alveolar macrophage MH-S cells after Pa infection using an autophagy based RT^2^ Profiler PCR Arrays (catalogue number: PAMM-084Z, Qiagen, Valencia, CA). The array analysis revealed that many autophagy related mRNAs (i.e., LC3-II, Atg4C, and Atg16L2) were upregulated in macrophages (S1A and S1B Fig, S1 Table), suggesting that autophagy may be involved in bacterial infection. To dissect whether the critical E1 enzyme Atg7 was required for host defense against Pa, we targeted Atg7 by siRNA in MH-S cells or isolated primary AM from wild type (WT) and *atg7* knockout (*atg7* ^-/-^) mice (S2A Fig). Phagocytosis or clearance assays were performed after shorter (1 h) or longer (12 h) periods of Pa infection [19]. Reduction or depletion of Atg7 showed decreased cell viability and reduced bactericidal activity upon Pa infection (S2B and S2C Fig). Importantly, inhibition of autophagy by 3-MA treatment decreased bacterial killing by macrophages without affecting the cell viability, thus resulting in bacterial evasion from immune response (S2D and S2E Fig), whereas rapamycin (Rapa, autophagy inducer) increased bactericidal activity upon infection (S2D and S2E Fig). These results imply that autophagy may facilitate bacterial clearance in macrophages upon Pa infection.

**Fig 1:**
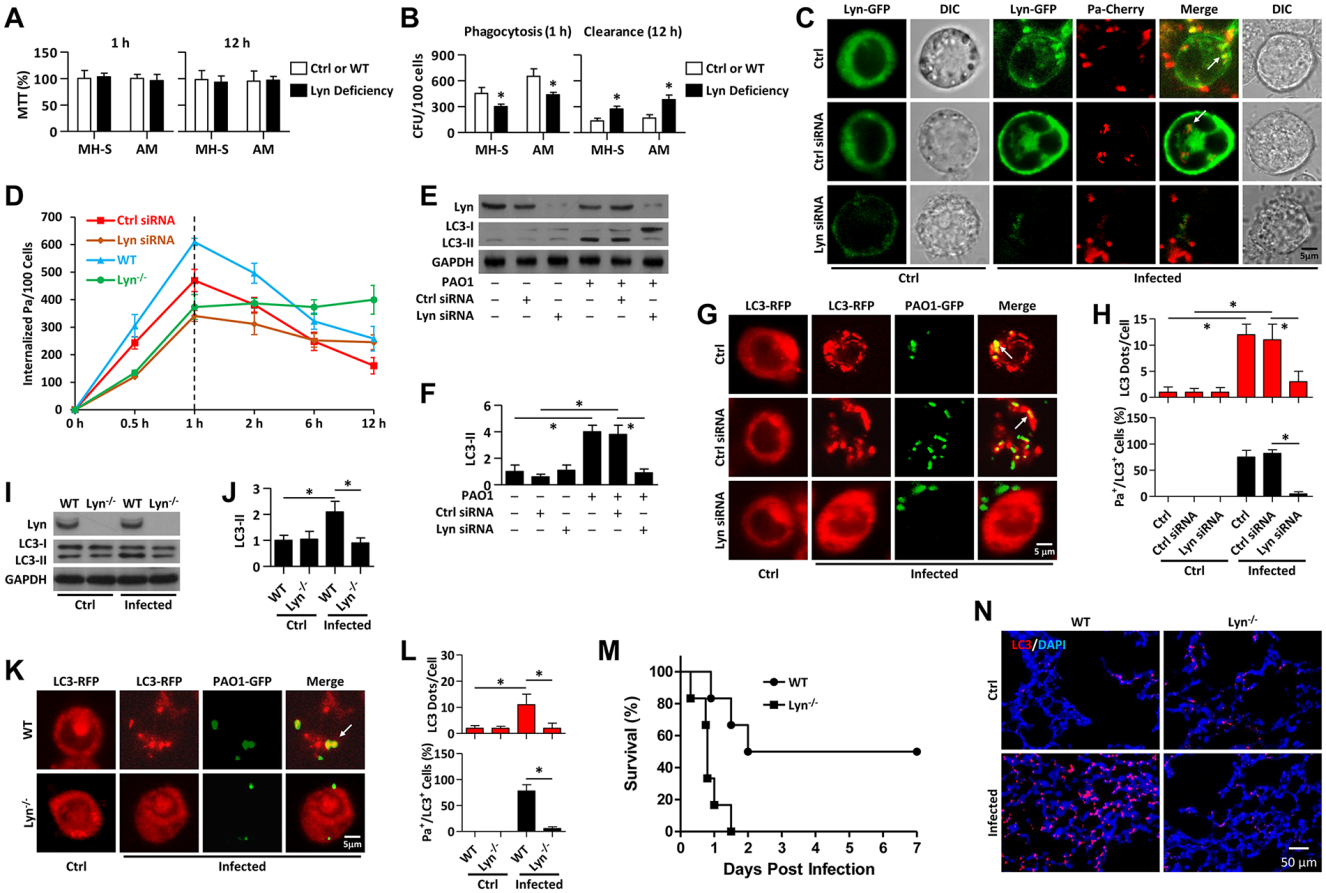
Lyn links phagocytosis to autophagy. (A, B) MH-S cells were transfected with either control (Ctrl) or Lyn siRNA at 10 nM for 24 h, respectively. Mouse primary AM from WT or Lyn^-/-^ mice were collected by bronchoalveolar lavage (BAL). Cells above were infected with PAO1 (MOI = 10, 1 h). Cell viability and phagocytosis assays were performed by MTT assays and by counting the number of live bacteria by plating colony forming units (CFU), respectively. After Pa infection (1 h), the cells were washed and then treated with polymyxin B (1 h). After 10 h, cell viability and clearance assays were performed as above. (C) MH-S cells were co-transfected with Ctrl or Lyn siRNA (10 nM) along with Lyn-GFP plasmid. After 24 h, the cells were infected with Pa-cherry (MOI = 10, 1 h). The colocalization or internalization was monitored using confocal microscopy. Scale bar = 5 μm. (D) Quantification of Fig 1C over time (100 cells each sample; at 1 h post-infection, polymyxin B were added for another 1 h and then removed) is shown as means±SD. (E) MH-S cells were transfected with Ctrl or Lyn siRNA respectively for 24 h and then infected with PAO1 (MOI = 10, 1 h). Cell lysates were performed for immunoblotting of Lyn and LC3. (F) Quantification of LC3-II expression in Fig 1E is shown. (G) The LC3-RFP plasmid was transfected into MH-S cells along with Ctrl or Lyn siRNA for 24 h. Cells were then infected with PAO1-GFP (MOI = 10, 1 h). Confocal microscopy images were taken to determine LC3 puncta staining upon infection. Scale bar = 5 μm. (H) LC3 puncta in each cell were counted and the percentage of LC3^+^/Pa^+^ events (cell with at least one LC3 puncta was considered to be positive) is shown. Data are derived from 100 cells in each sample. (I) Primary AM from WT or Lyn^-/-^ mice were collected. Cells were infected with PAO1 (MOI = 10, 1 h), and cell lysates were collected and performed for immunoblotting of Lyn and LC3. (J) Quantification of LC3-II expression in Fig 1I is shown. (K) Primary AM were transfected with LC3-RFP for 24 h and infected with PAO1-GFP (MOI = 10, 1 h). Confocal microscopy images show significant LC3 puncta upon Pa infection. Scale bar = 5 μm. (L) Quantifications of LC3 puncta in each cell and the percentage of LC3^+^/Pa^+^ events are shown as above. (M) WT mice and Lyn^-/-^ mice were infected with 1×10^7^ CFU of PAO1 (6 mice/group). Survival test was represented by Kaplan-Meier survival curves (p = 0.0065; 95% confidence interval, 0.8–4.5; Log-rank Test). (N) Lungs from above were removed and paraffin sectioned for immunostaining of LC3. Scale bar = 50 μm. The data represent means+SD from three experiments. One-way ANOVA (Tukey’s post hoc); *, p<0.05.

A prior study suggested that Lyn may be a critical factor during Pa infection [17]. MH-S cells and mouse primary AM were used to assess phagocytosis and bacterial clearance using CFU assays. After Lyn knockdown, the cell viability was determined using a MTT assay, which showed no difference upon Pa infection (Fig 1A), while bactericidal activity was impaired by Lyn deficiency (Fig 1B). Next, we transfected MH-S cells with Lyn-GFP, scrambled siRNA or Lyn siRNA, and infected the cells with another fluorescent-emitting and lipid-affinitive strain, Pa-Cherry. We observed significant Lyn aggregates (puncta) around the invading Pa by confocal laser scanning microscopy (CLSM) (Fig 1C). CLSM imaging demonstrated that internalization by macrophages was decreased with Lyn deficiency at the early time, suggesting the involvement of Lyn in phagocytosis (Fig 1D). After removing the floating and surface bacteria by polymyxin B treatment at 1 h, the internalized Pa was killed with time. Lyn deficient AM showed decreased bactericidal ability (Fig 1D). Endogenous LC3 conversion into phosphatidylethanolamine-conjugated LC3-II was drastically inhibited by Lyn siRNA in MH-S cells as determined by immunoblotting (Fig 1E and 1F). Further, Lyn knockdown impeded LC3 puncta formation (Fig 1G and 1H). To reflect the physiological relevance, we also isolated primary AM to determine whether Lyn regulates autophagy in these cells. Similarly, significantly-decreased LC3 conversion was found in primary AM from Lyn^-/-^ mice upon Pa infection (Fig 1I and 1J); and primary AM from Lyn^-/-^ mice also exhibited a marked reduction in LC3 puncta as detected by CLSM imaging (Fig 1K and 1L). MLE-12 cells, a murine lung epithelial cell line that does not appreciably phagocytose or clear bacteria, also showed LC3 puncta upon Pa infection, which was also impaired by Lyn deficiency or by perturbing autophagy (3-MA addition) (S2F Fig). *In vivo* experiments were then used to determine autophagy induction upon Pa infection. Lyn^-/-^ mice exhibited increased mortality as compared to WT mice after Pa infection (Fig 1M). The increased bacterial burdens in the lungs also implied severely impaired bacterial killing in Lyn^-/-^ mice (S2G Fig). Lung histological analysis revealed that induction of LC3 puncta was decreased (Fig 1N), and pathophysiological tissue damage was more severe in Lyn deficiency following Pa infection (S2H Fig). Since PMN play an important role in innate immunity to Pa infection [20], PMN migration into infected lungs was measured and found to be increased in Lyn^-/-^ mice as determined by immunostaining with Ly6G (S2I Fig). These results indicate that Lyn is involved in an autophagy related phagocytosis to benefit Pa clearance in macrophages, which may preserve host cell viability after infection.

### Lyn is required for autophagy induction upon Pa infection

**Fig 2:**
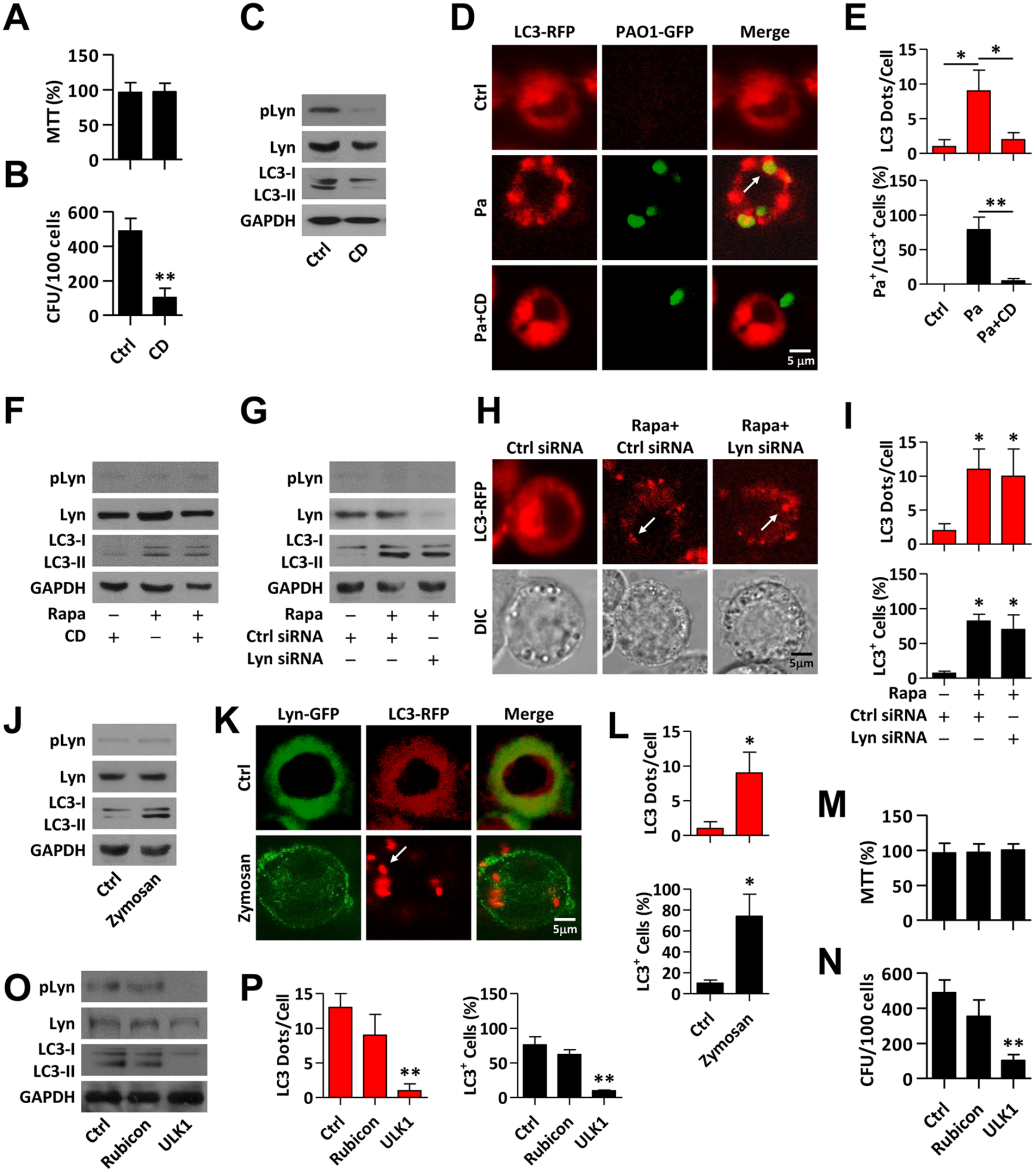
Lyn plays an important role in recruitment of LC3 upon Pa infection. (A) MH-S cells were pretreated with CD (2.5 μg/ml, 30 min), and then infected with PAO1 (MOI = 10, 1 h). Cell viability was tested by MTT assay. (B) Cells treated as above were lysed for CFU assay. (C) Cells lysates were performed for immunoblotting of pLyn, Lyn and LC3. (D, E) MH-S cells were transfected with LC3-RFP plasmid for 24 h and pretreated with CD (2.5 μg/ml, 30 min). The cells were then infected with PAO1-GFP (MOI = 10, 1 h). Confocal microscopy images were used to detect LC3 puncta upon Pa infection. LC3 puncta in each cell were counted and the percentage of LC3^+^/Pa^+^ colocalization (LC3-RFP and PAO1-GFP) is shown. Data are derived from 100 cells in each sample. (F) MH-S were pretreated with CD (2.5 μg/ml, 30 min) or rapamycin (500 nM, 12 h) and cells were lysed for immunoblotting of pLyn, Lyn and LC3. (G) MH-S cells were transfected with Ctrl or Lyn siRNA for 24 h, and treated with rapamycin (500 nM, 12 h). Cells lysates were performed for immunoblotting of pLyn, Lyn and LC3. (H, I) MH-S cells were co-transfected with LC3-RFP and Ctrl or Lyn siRNA for 24 h. The cells were treated with rapamycin (500 nM, 12 h). Confocal microscopy images were used to show LC3 puncta. LC3 puncta in each cell were counted. The percentage of LC3^+^ events (cell with more than five LC3 puncta was considered as positive) is shown. Data are derived from 100 cells in each sample. (J) MH-S cells were treated with Zymosan (10 μg/ml, 1 h). Cell lysates were performed for immunoblotting of pLyn, Lyn and LC3. (K) MH-S cells were co-transfected with Lyn-GFP and LC3-RFP for 24 h. The cells were treated with Zymosan (10 μg/ml, 1 h). Confocal microscopy images were used to show Lyn or LC3 puncta. (L) LC3 puncta in each cell were counted, and the percentage of LC3^+^ events is shown as above. Data are derived from 100 cells in each sample. (M, N) MH-S cells were transfected with Ctrl, Rubicon or ULK1 siRNA for 24 h. The cells were infected with PAO1 (MOI = 10, 1 h). Cell viability and phagocytic abilities were tested by MTT or CFU assays as above. (O) Cell lysates from above were used for immunoblotting to detect pLyn, Lyn and LC3, respectively. (P) MH-S cells were co-transfected with Ctrl, Rubicon or ULK1 siRNA, respectively along with LC3-RFP for 24 h. The cells were infected with PAO1-GFP (MOI = 10, 1 h). LC3 puncta in each cell were counted, and the percentage of LC3^+^ events is shown as above. Data are derived from 100 cells in each sample. All data are representative as means+SD of three independent experiments. One-way ANOVA (Tukey’s post hoc); *, p<0.05; **, p<0.01. Scale bar = 5 μm.

To distinguish whether Lyn plays a role in Pa infection-induced autophagy, we first blocked internalization of Pa into macrophages using cytochalasin D (CD), a phagocytosis inhibitor. While CD had no effect on cell viability upon Pa infection (Fig 2A), its addition inhibited phagocytosis by macrophages (Fig 2B). Interestingly, CD decreased LC3 conversion and phosphorylation (Tyr297) of Lyn (pLyn) upon Pa infection (Figs 2C and S3A). CLSM imaging also indicated that CD decreased LC3 puncta upon Pa infection (Fig 2D and 2E). To define the relationship of CD with autophagy, rapamycin was used as a positive control to induce autophagy. CD did not induce LC3 conversion or phosphorylation of Lyn. Moreover, CD did not inhibit rapamycin-induced LC3 conversion (Figs 2F and S3B). Next, siRNA interference was used to further determine the role of Lyn in autophagy. As shown in Figs 2G and S3C, Lyn deficiency did not affect rapamycin-induced autophagy without infection. CLSM imaging also confirmed that Lyn deficiency did not affect rapamycin-induced LC3 puncta (Fig 2H and 2I). These data suggest that Lyn is required for Pa infection-induced autophagy. We hypothesized that Lyn-mediated phagocytosis is associated with autophagy upon Pa infection. Zymosan (yeast wall extract) was used to treat macrophages, which did not alter cell viability (S3D Fig). LC3 conversion was found to be induced by Zymosan treatment. However, Zymosan did not induce Lyn phosphorylation (Fig 2J and S3E Fig). In addition, CLSM imaging showed LC3 puncta formation while no Lyn puncta were formed upon Zymosan treatment of macrophages (Fig 2K and 2L). Furthermore, Gram-positive bacterium *S*. *pyogenes* (Sp) infection was used to determine the induction of LC3 puncta and Lyn puncta by CLSM imaging (S3F Fig). Collectively, these data indicated the role of Lyn in bacterial infection-induced autophagy. To distinguish conventional autophagy from LAP, the upstream preinitiation complex (ULK1/2, which is required for autophagy but not LAP), and Rubicon (which mediates LAP but not autophagy) were knocked down by siRNA interference strategy, respectively (S3G Fig) [21]. Knockdown of Rubicon does not affect the cell viability upon Pa infection (Fig 2M); however, ULK1 interference resulted in decreased bacterial phagocytosis by macrophages (Fig 2N). In addition, ULK1 interference led to decreased pLyn level and also LC3 lipidation as compared to Ctrl siRNA transfected cells (Figs 2O and S3H). CLSM imaging quantitation also showed decreased LC3 puncta by ULK1 deficiency upon Pa infection (Fig 2P). However, Rubicon interference did not affect Lyn phosphorylation while slightly decreased LC3 lipidation upon Pa infection, with only limited phagocytosis reduction, indicating that LAP is less relevant to this infection model (Fig 2M–2P, S3H Fig). To further determine that LC3-lipidation and dot formation occur upon Pa infection, chloroquine was used to inhibit lysosome activities. Lyn phosphorylation was found with or without chloroquine. Furthermore, we observed that LC3-II conversion had accumulated with chloroquine treatment upon Pa infection determined by immunoblotting (S3I and S3J Fig).

### Lyn kinase activities are needed to facilitate infection-induced autophagy

**Fig 3:**
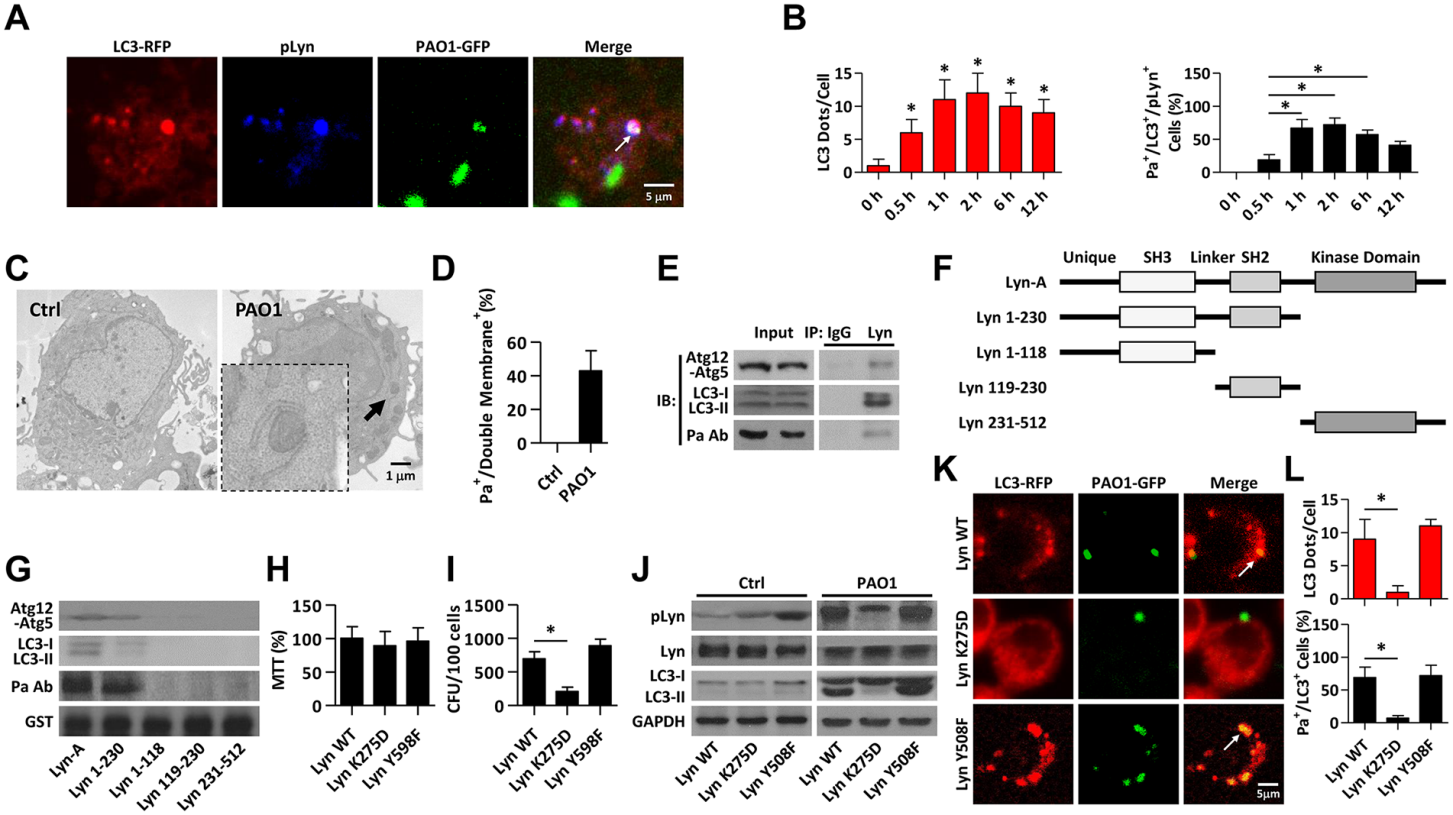
Lyn kinase activities are required to facilitate autophagy related phagocytosis. (A) MH-S cells were transiently transfected with LC3-RFP. After 24 hours cells were infected with PAO1-GFP (MOI = 10, 1 h). Cells were fixed and stained with anti-pLyn antibody for immunofluorescence detection. Arrows indicate significant LC3 puncta colocalized with pLyn and Pa using CLSM imaging. Scale bar = 5 μm. (B) The statistic results from samples in A show LC3 puncta in each cell that are infected with Pa for different time. At 1 h post-infection, polymyxin B was added (1 h) to kill residual bacteria. The percentage of LC3^+^/Pa^+^/pLyn^+^ events (cell with colocalized puncta of LC3-RFP, PAO1-GFP, and pLyn) is shown. Data are derived from 100 cells in each sample. (C, D) MH-S cells were infected with PAO1 (MOI = 10, 1 h). After infection, cells were processed and examined by TEM. Arrow indicates autophagosome with double membranes containing internalized bacteria. The percentage of internalized Pa surrounded by double membrane is shown. Data are from 20 cells in each sample. Scale bar = 1 μm. (E) MH-S cells were infected with PAO1 as above. After infection, cell lysates were processed for co-IP to examine the interactions between Lyn and Atg12-Atg5, LC3 and Pa. (F) GST tagged Lyn peptide fragments were used to study *in vitro* association of Lyn with autophagy related proteins. (G) MH-S cells were infected with PAO1 (MOI = 10, 1 h) and then lysed for pull-down assay. GST-Lyn 1–230 containing both SH3 and SH2 domains shows association with Atg5-Atg12, LC3 and Pa. (H, I) MH-S cells were transfected with Lyn WT, DN (Lyn K275D), and constitutively active (Lyn Y598F) plasmid for 24 h and then treated with PAO1 as above. MTT assays were used to assess cell viability. CFU assays were used to measure phagocytosis. (J) Cells above were lysed for immunoblotting to detect the protein levels of pLyn, Lyn and LC3. (K, L) MH-S cells were transfected with Lyn WT, DN, and constitutively active plasmid for 24 h and then treated with PAO1-GFP (MOI = 10, 1 h). Confocal microscopy images were used to show significant LC3 puncta staining upon Pa infection. LC3 puncta in each cell were counted, and the percentage of LC3^+^/Pa^+^ events (cell with colocalized puncta of LC3-RFP and PAO1-GFP) is shown. Data are derived from 100 cells in each sample. Scale bar = 5 μm. All data are representative as means+SD of three independent experiments. One-way ANOVA (Tukey’s post hoc); *, p<0.05; **, p<0.01.

Interestingly, CLSM imaging showed that LC3 recruitment and phosphorylation of Lyn localized together with invading Pa (Fig 3A and 3B). To unravel whether the phagocytosis of Pa is involved in autophagosome formation, we detected newly formed autophagosomes using transmission electron microscopy (TEM) on Pa-infected MH-S cells. Pa-containing autophagosomes with double membranes were identified by TEM (Fig 3C and 3D), providing additional evidence that Pa-containing autophagosomes had formed, which is also termed as xenophagy. Next, co-immunoprecipitation (co-IP) was performed to determine the interaction of Lyn with autophagy-related proteins. As shown in Fig 3E, we observed that Lyn could bind well to Atg12-Atg5 and LC3 complex upon Pa infection. In addition, we found that Lyn associated with Pa (Fig 3E), which suggested that Lyn may play a role in regulating autophagosome formation upon bacterial infection. To further understand the molecular mechanism in Lyn-mediated autophagosome formation, we asked which domain(s) of Lyn is required for interaction with the functionally or structurally relevant proteins. Lyn-GST peptides with distinct functional domains (see schematic, Fig 3F) were prepared as described previously [16,22]. The Lyn-GST fragments were coated on immobilized glutathione agarose beads to pull-down interacting partners from MH-S cell lysates and probed for Atg12-Atg5, LC3, or Pa, respectively. The presence of both Src homology 2 (SH2) and SH3 domains of Lyn were found to be required for interaction with signaling proteins within autophagosomes, whereas the kinase domain was dispensable for the interaction upon Pa infection (Fig 3G, S4A and S4B Fig). These data further suggested that Lyn may interact with critical autophagic proteins localized in autophagosomes through SH2 and SH3 binding regions.

We next investigated whether this interaction specifically occurred in macrophages with strong phagocytosis. Primary AM and epithelial cells (MLE-12) were infected with Pa and cell lysates were applied for pull-down by Lyn-GST fragments. Although SH2 and SH3 domains of Lyn were required for interactions with autophagic proteins and binding to Pa in macrophages, no apparent Lyn-Pa interaction in MLE-12 cells was observed (S4C and S4D Fig), which may be due to different mechanisms of uptaking Pa by epithelial cells. To confirm that Lyn activation was responsible for the underlying process, MH-S cells were transfected with Lyn WT, Lyn K275D (dominant negative), and Lyn Y508F (constitutively active) constructs and treated with Pa. The transfection of different plasmids had no effect on the cell viability upon infection (Fig 3H), whereas Lyn K275D transfected cells showed impaired phagocytic ability upon bacterial infection (Fig 3I). Lyn activation was detected by immunoblotting for pLyn. While Lyn Y508F plasmid-transfected cells showed enhanced activity of Lyn kinase, Lyn K275D-transfected cells showed decreased activity as compared to Lyn WT-transfected cells (Figs 3J and S4E). LC3-II conversion was found to be inhibited in Lyn K275D transfected cells upon Pa infection (Figs 3J and S4E). Furthermore, LC3 puncta revealed that Lyn kinase activities were well correlated with Pa-induced autophagy in macrophages (Fig 3K and 3L). Our data also showed that PP2 (a commonly used Lyn inhibitor) reduced infection-induced autophagy (S4F and S4G Fig). Overall, the results demonstrated that the role of Lyn in infection-induced autophagy was dependent on its kinase activities.

### TLR2 is involved in Lyn-initiated autophagy upon Pa infection

**Fig 4:**
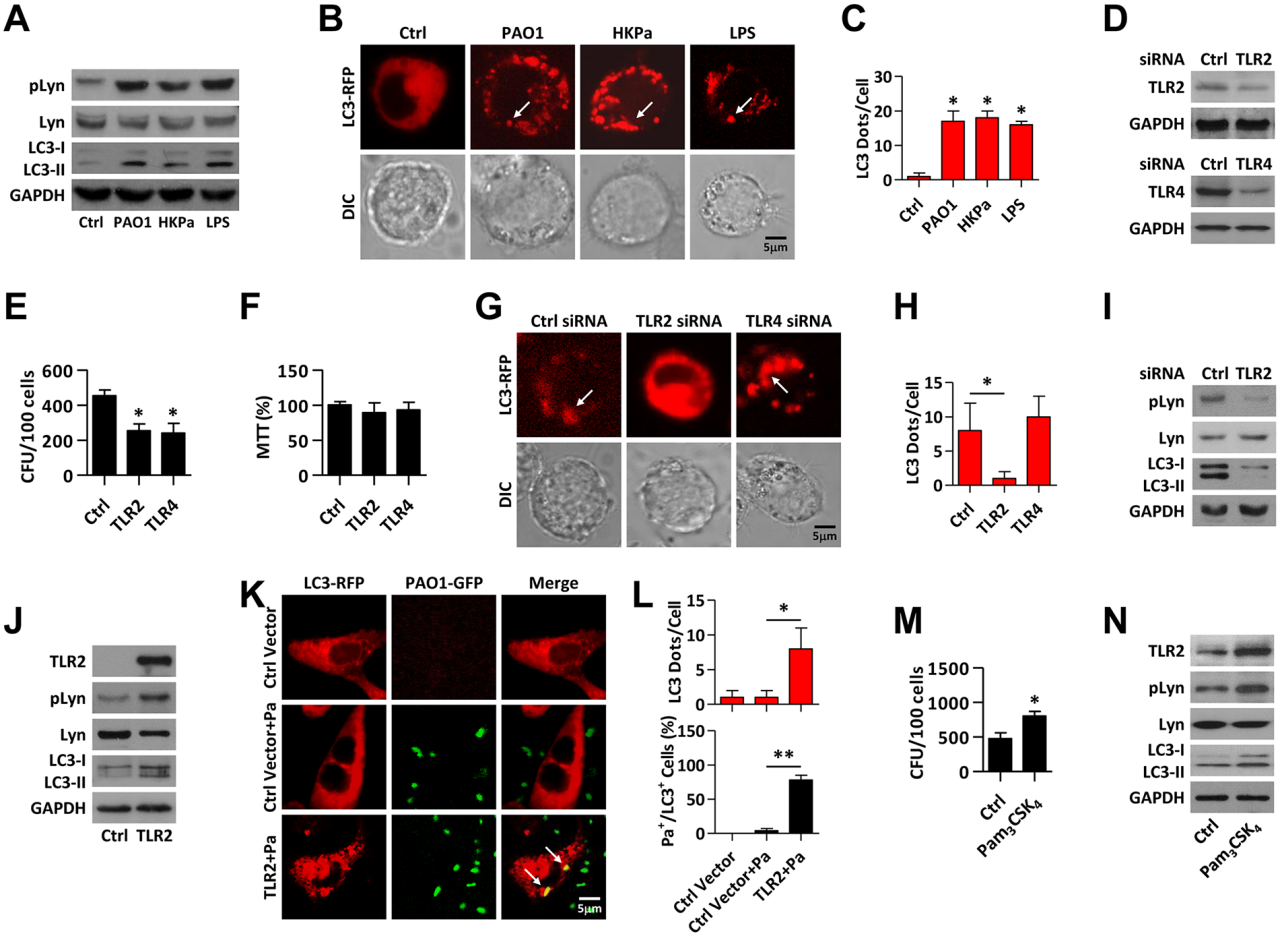
TLR2 is involved in infection-induced autophagy. (A) MH-S cells were infected by PAO1 (MOI = 10), HKPa (MOI = 10) and Pa LPS (100 ng/ml) for 1 h. HKPa was obtained by heating PAO1 at 60°C for 60 min. Cell lysates were collected and immunoblotting of pLyn, Lyn and LC3 were performed. (B, C) MH-S cells were transfected with LC3-RFP plasmid for 24 h. CLSM imaging showed infection by PAO1, HKPa and Pa LPS induced LC3 puncta. LC3 puncta in each cell were counted. Data are derived from 100 cells in each sample. (D) MH-S cells were transfected with Ctrl, TLR2 or TLR4 siRNA for 24 h, respectively. Immunoblotting were performed to prove the knock down of TLR2 or TLR4. (E, F) After TLR2 or TLR4 knock down as above, the cells were infected with PAO1 (MOI = 10, 1 h). MTT assays were used to measure the cell viability and CFU assays were used to measure phagocytosis. (G, H) MH-S cells were co-transfected with LC3-RFP and Ctrl, TLR2 or TLR4 siRNA for 24 h, respectively. Cells were then infected with PAO1 as above. CLSM imaging showed infection-induced LC3 puncta. LC3 puncta in each cell were counted. Data are derived from 100 cells in each sample. (I) MH-S cells were transfected with Ctrl or TLR2 siRNA for 24 h, respectively. Cells were infected with PAO1 as above. Cell lysates were performed for immunoblotting to detect pLyn, Lyn and LC3. (J) The HEK293 cells were stably transfected with a pUNO-TLR2 plasmid which expresses TLR2. TLR2 stable expression cells and 293/null cells were infected with PAO1 as above. Immunoblotting was performed to detect TLR2, pLyn, Lyn and LC3. (K, L) 293 cells were transiently transfected with LC3-RFP. After 24 hours cells were infected with PAO1-GFP (MOI = 10, 1 h). Arrows indicate significant LC3 puncta upon Pa infection using CLSM imaging. LC3 puncta in each cell were counted and the percentage of LC3^+^/Pa^+^ events (cell with colocalized puncta of LC3-RFP and PAO1-GFP) is shown. Data are derived from 100 cells in each sample. (M, N) MH-S cells were pretreated with Pam_3_CSK_4_ (300 ng/ml), and infected with PAO1 as above. Cells were homogenized for CFU and immunoblotting to detect TLR2, pLyn, Lyn and LC3. All data are representative as means+SD of three independent experiments. One-way ANOVA (Tukey’s post hoc); *, p<0.05; **, p<0.01. Scale bar = 5 μm.

We next attempted to determine whether a single bacterial component (i.e. LPS), can induce autophagy. Interestingly, heat-killed Pa (HKPa) and LPS all caused LC3-II conversion to a similar extent as Pa, as detected by immunoblotting (Figs 4A and S5A). On the other hand, CLSM fluorescence microscopy showed that HKPa or LPS also induced LC3 puncta (Fig 4B and 4C). Importantly, we also observed increased phosphorylation of Lyn by LPS pretreatment (Figs 4A and S5A). These results indicate that Pa or LPS is potentially sufficient to induce autophagy in macrophages. Based on our previous observations [17], we probed the role of TLR2 and TLR4 as upstream signals to initiate autophagy. After TLR2 or TLR4 knockdown (Fig 4D), MH-S cells were infected with Pa. Both TLR2 and TLR4 deficiency impaired the phagocytosis of Pa (Fig 4E), while cell viability was not altered (Fig 4F). However, only TLR2 knockdown showed impaired LC3 puncta formation upon bacterial infection (Fig 4G and 4H). Immunoblotting also showed that TLR2 deficiency inhibited LC3 conversion and Lyn phosphorylation upon bacterial infection (Figs 4I and S5B). These results suggest that TLR2 may play a role in autophagy induction during Pa infection (although other TLRs’ role cannot be excluded at this time). To delve into the involvement of TLR2, HEK293 cells with stable TLR2 expression were infected by Pa. Although these cells are not phagocytes, Lyn activation (shown as pLyn level) or autophagy induction (shown as LC3 conversion or LC3 puncta) was much stronger in cells overexpressing TLR2 than vector control cells (Fig 4J–4L, S5C Fig). Pam_3_CSK_4_, a TLR2 agonist, increased phagocytosis of Pa (Figs 4M and S5D); however, Pam_3_CSK_4_ had no impact on LC3 puncta if left uninfected (S5E Fig). Pam_3_CSK_4_ also increased Lyn activation or autophagy in MH-S cells upon Pa infection as determined by immunoblotting (Figs 4N and S5F). These results suggested that Lyn may serve as a bridge between TLR2 and autophagy after Pa infection.

### Autophagy plays a role in Lyn mediated bacterial clearance

**Fig 5:**
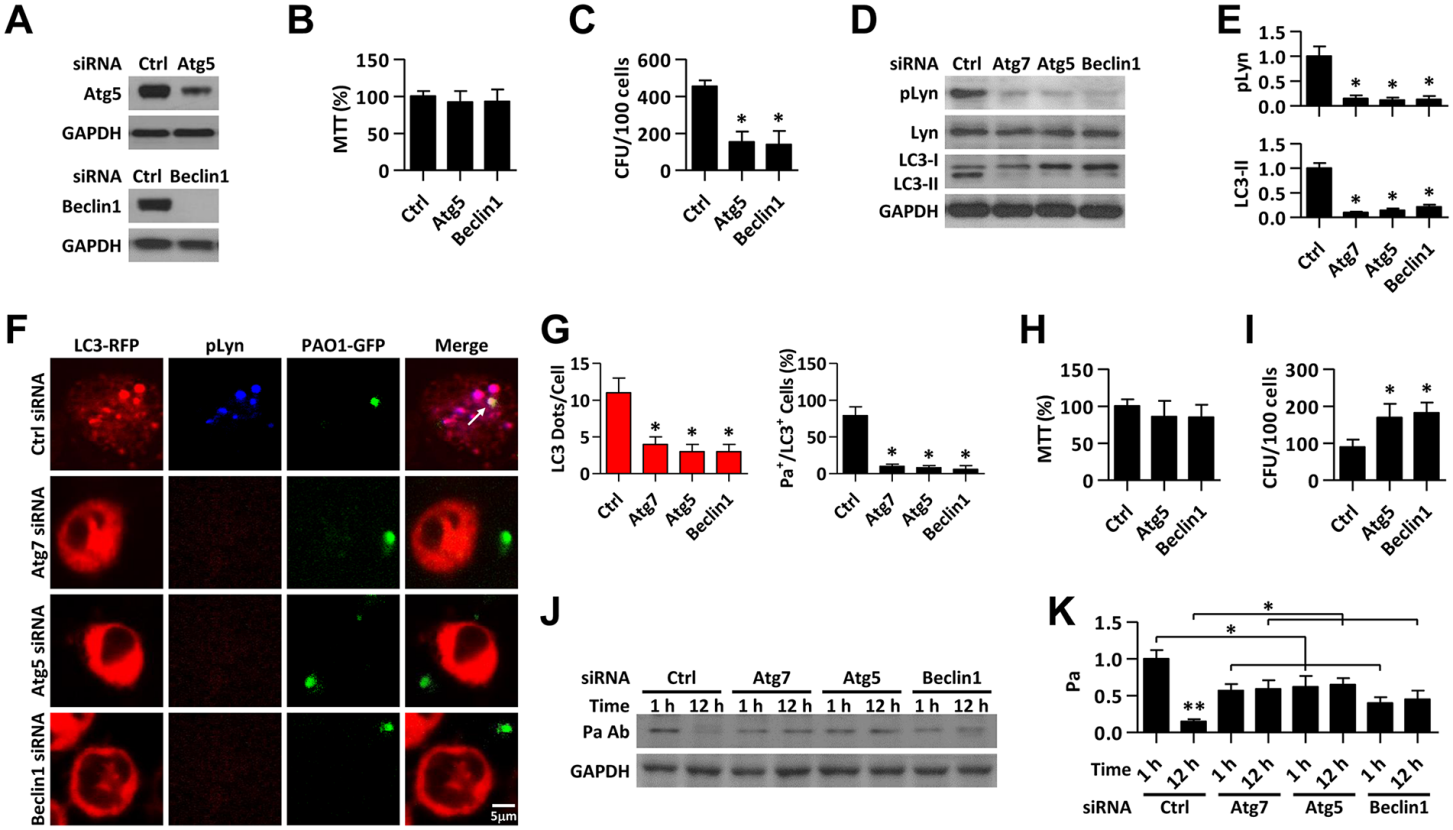
Autophagy is important for Lyn-dependent elimination of Pa in macrophages. (A) MH-S cells were transfected with Ctrl, Atg5 or Beclin1 siRNA for 24 h, respectively. Immunoblotting was performed to verify the knock down of these two genes. (B) After Atg5 or Beclin1 was knocked down, MTT assay was used to determine the cell viability upon PAO1 infection (MOI = 10, 1 h). (C) CFU assay was performed to test the phagocytosis ability from above samples. (D) Cells above were collected and lysed for immunoblotting to measure the phosphorylation level of Lyn. (E) Quantification of pLyn and LC3-II level in D is shown. (F) MH-S cells were transfected with LC3-RFP for 24 h. Cells were infected with PAO1-GFP (MOI = 10, 1 h). Immunostaining was used to detect pLyn. Pa surrounded by LC3 and pLyn was detected by confocal microscope. Scale bar = 5 μm. (G) LC3 puncta in each cell were counted. The percentage of LC3^+^/Pa^+^/pLyn^+^ events (cell with colocalized puncta of LC3-RFP, PAO1-GFP, and pLyn) is shown. Data are derived from 100 cells in each sample. (H) MH-S cells were infected with PAO1 as above. After 1 h, cells were washed and subjected to polymyxin B for another 1 h. 10 h later, cell viability was determined by MTT assay. (I) Clearance assays were performed by counting CFU. (J) MH-S cells were infected with PAO1 for 1 h or 12 h as above. Cell lysates were performed for immunoblotting to test the invaded bacteria using Pa antibody. (K) Quantification of Pa protein in J is shown. All data are representative as means+SD of three independent experiments. One-way ANOVA (Tukey’s post hoc); *, p<0.05; **, p<0.01.

To assess whether autophagy has a role on Lyn-dependent elimination of Pa in macrophages, we knocked down Atg5 and Beclin1 using siRNA (Fig 5A). Atg5 or Beclin1 has little effect on cell viability upon Pa infection (Fig 5B); however, phagocytosis was inhibited by down-regulating these two genes (Fig 5C). Immunoblotting also showed that the phosphorylation of Lyn was decreased by Atg7, Atg5 or Beclin1 deficiency (Fig 5D and 5E). CLSM imaging also showed the LC3 puncta and pLyn level was dampened by a deficiency in either of these autophagic genes (Fig 5F and 5G). The clearance of phagocytized bacteria was also dampened by inhibiting Atg5 or Beclin1 in AMs upon Pa infection (Fig 5H and 5I). We then performed immunoblotting to verify the digestion of the invading bacteria and found that when autophagic genes were knocked down, the degradation of the bacteria proteins was blocked over time (Fig 5J and 5K). All these results suggest that autophagy plays an important role in Lyn-mediated phagocytosis and clearance upon bacterial infection.

### Lyn regulates infection-induced autophagy through Rab and cofilin

**Fig 6:**
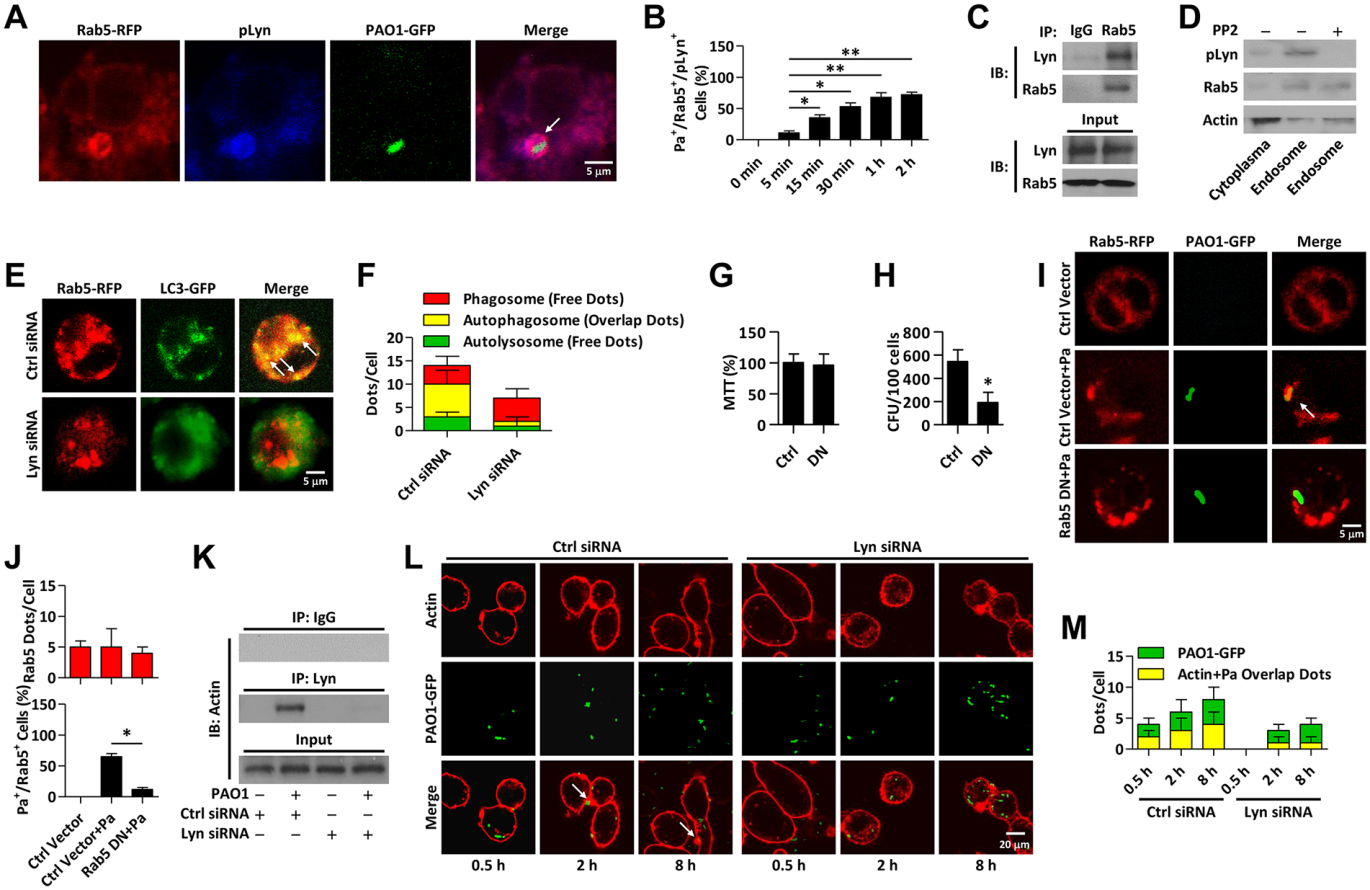
Lyn regulate infection-induced autophagy through Rab5. (A) MH-S cells were transfected with Rab5-RFP for 24 h. Cells were infected with PAO1-GFP (MOI = 10, 1 h). Immunostaining was used to detect pLyn. Pa surrounded by Rab5 and pLyn was detected by confocal microscope. Scale bar = 5 μm. (B) The percentage of Rab5^+^/Pa^+^/pLyn^+^ colocalization (Rab5-RFP, PAO1-GFP, and pLyn puncta) is shown. Data are derived from 100 cells in each group. (C) MH-S cells were infected with PAO1 (MOI = 10, 1 h). Co-IP was performed to detect the protein interactions between Lyn and Rab5. (D) MH-S cells were pretreated with PP2 (5 nM, 30 min). Phagosomes were isolated and lysed for immunoblotting to detect pLyn and Rab5. (E, F) MH-S cells were co-transfected with Rab5-RFP and LC3-GFP as well as Lyn siRNA for 24 h, respectively. Cells were infected with PAO1 as above. CLSM imaging showed the localization (different puncta). Data are derived from 100 cells for each sample. Scale bar = 5 μm. (G, H) MH-S cells were transfected with Rab5-RFP or Rab5-DN-RFP plasmid for 24 h. Cells were infected with PAO1-GFP as above. Cell viability was determined using MTT assay and phagocytosis was performed by CFU assay. (I, J) CLSM imaging showed the localization of Rab5 and Pa. Scale bar = 5 μm. Rab5 puncta were counted in each cell. The percentage of Rab5^+^/Pa^+^ events (cell with colocalized puncta of Rab5-RFP and PAO1-GFP) is shown. Data are derived from 100 cells in each group. (K) MH-S cells were transfected with Ctrl or Lyn siRNA for 24 h, respectively. Whole cell lysates were immunoprecipitated (IP) with beads coated with Lyn antibody and immunoblotted with actin antibody. (L, M) Cells were infected with PAO1-GFP (MOI = 10) for different time. Polymerized actin was stained with rhodamine-phalloidin. Confocal microscopy showing rhodamine-phalloidin staining (red) around GFP-expressing Pa. Phagosomes containing degraded bacteria are heavily labeled for polymerized actin (white arrowheads). Scale bar = 20 μm. Internalized Pa and colocalized puncta were counted in each cell. Data are derived from 100 cells in each group. One-way ANOVA (Tukey’s post hoc); *, p<0.05.

To further assess the phagocytosis process, we elucidated whether Rab5 (early endosome marker) is involved in Lyn-mediated phagocytosis and subsequent bacterial clearance. We transfected MH-S cells with Rab5-RFP plasmid and infected the cells with Pa for different times. Immunostaining was next performed to probe pLyn to evaluate the internalized bacteria using CLSM microscopy. The number of phagocytized Pa was increased at the early time of infection. Interestingly, Rab5 and Lyn were both found to be surrounded with invading Pa, which implied that Rab5 may play a role in conjunction with Lyn in phagocytosis (Fig 6A and 6B). Next, co-IP was performed to confirm the interaction of Lyn and Rab5 (Figs 6C and S6A). To this end, we isolated phagosomes after Pa infection and the results showed that pLyn was found in the same fraction as Rab5 (e.g., phagosomes), while PP2 pretreatment impaired this process (Fig 6D). To test our hypothesis that Lyn is involved in Rab5-mediated autophagy related phagocytosis of Pa, we co-transfected Rab5-RPF, LC3-GFP with Ctrl or Lyn siRNA into MH-S cells. After Lyn knockdown, the formation of autophagosomes was impaired upon Pa infection (Fig 6E and 6F). Similar results were found with Rab7 (late endosome marker). We observed an interaction of Lyn with Rab7 (S6A Fig; Lyn deficiency also decreased autophagosome-lysosome formation upon Pa infection (S6B and S6C Fig). These data suggest that Lyn-mediated phagocytosis may be dependent on the conserved endocytosis pathway involving Rab families. We then used dominant negative plasmids to down-regulate Rab5 and Rab7 and found that Rab5-DN-RFP transfection did not affect cell viability (Fig 6G), while dampened the phagocytosis (Fig 6H, S6D and S6E Fig) and colocalization of Rab5 with Pa (Fig 6I and 6J). Importantly, Rab7-DN-RFP transfection also significantly inhibited phagocytosis and colocalization of Rab7 and Pa inside the autolysosome (S6F and S6G Fig). Collectively, these findings suggest that Lyn, together with Rab family members, participated in the transportation of invading bacteria in phagocytosis and clearance upon infection.

To determine whether Lyn is involved in actin remodeling in macrophages, we performed co-IP and found that Lyn interacted with actin after Pa infection (Fig 6K). Lyn siRNA-transfected cells showed significantly decreased polymerized F-actin indicated by phalloidin staining (around Pa containing phagosomes) vs. Ctrl siRNA-transfected cells at both 2 h and 8 h (Fig 6L and 6M). Also, flotillin-1 expression, which is required for nucleation of actin on phagosomal membrane, correlated with the internalization of Pa, and was inhibited by Lyn siRNA interference (S6H and S6I Fig). These findings prompted us to determine whether Pa-containing phagosomes are involved in polymerized actin, we evaluated the phosphorylation of cofilin, a conserved actin-modulating protein. Uninfected cells allowed the phosphorylation of basal amounts of cofilin, whereas Pa infection led to increased phosphorylation of cofilin. Further, cofilin remained unphosphorylated in Lyn-deficient macrophages following Pa infection (S6H and S6I Fig). Moreover, we found that Lyn interacted with cofilin after Pa infection as evaluated with co-IP (S6J Fig). Thus, interaction between actin and Lyn is required for modulation of the phosphorylation state of cofilin during Pa infection. Thus, Lyn is required for both F-actin network formation and phosphorylation of cofilin during Pa infection.

### Lyn mediates fusion of lysosome with Pa-containing autophagosome

**Fig 7:**
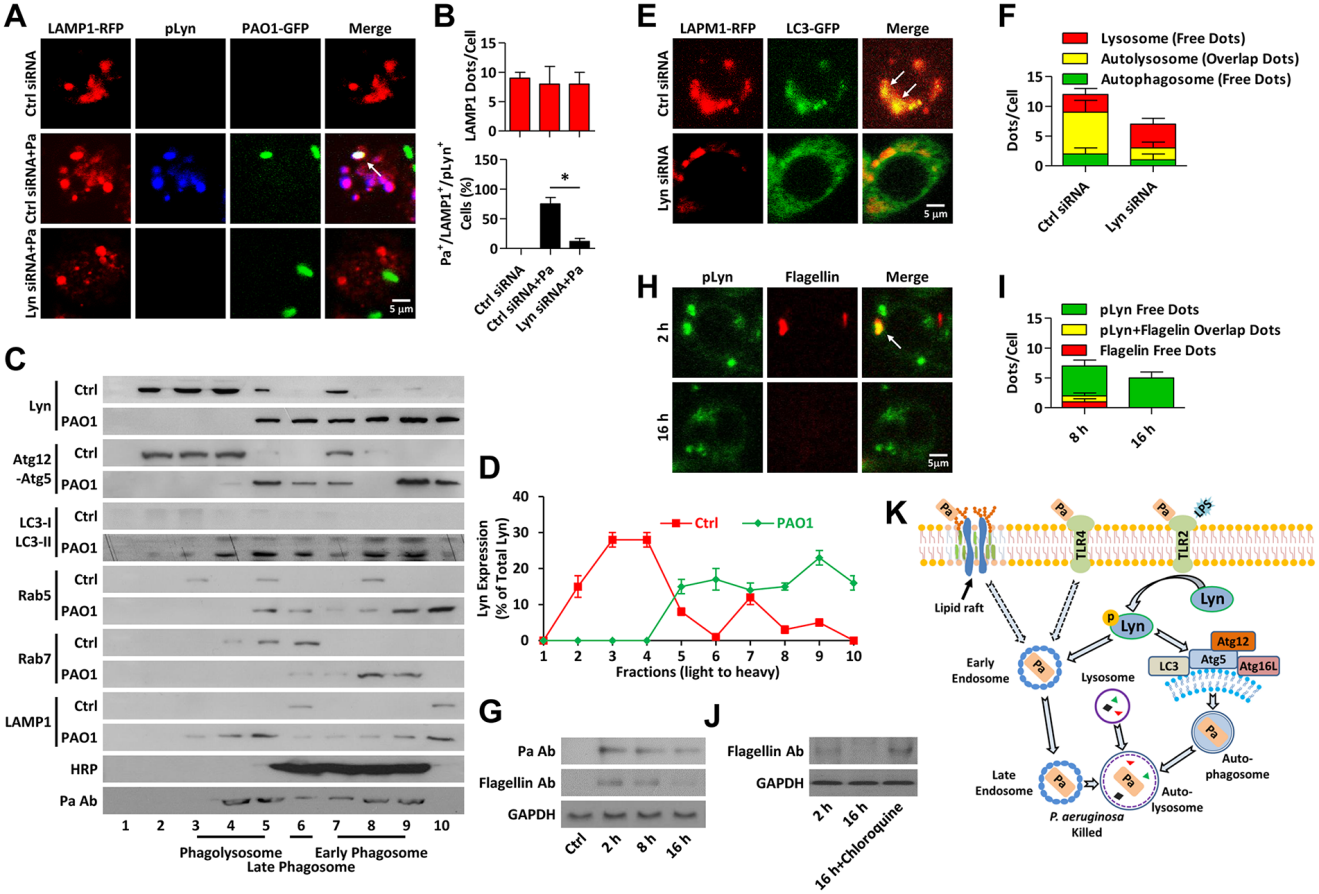
Lyn delivers bacteria to lysosome. (A, B) MH-S cells were transfected with LAMP1-RFP for 24 h. After infected with PAO1-GFP (MOI = 10, 8 h), immunostaining was used to detect pLyn. Three colors overlap was detected by confocal microscope. Arrows indicate the colocalized puncta. LAMP1 puncta in each cell were counted. The percentage of LAMP1^+^/Pa^+^/pLyn^+^ events (cell with colocalized puncta of LAMP1-RFP, PAO1-GFP, and pLyn) is shown. Data are derived from 100 cells in each sample. (C) MH-S cells were infected by PAO1 as above and lysed for phagosome isolation by the sucrose density gradient method without detergent to keep the intracellular membranes intact. Phagosome fractions were resolved by SDS-PAGE and analyzed by immunoblotting with Lyn, Atg12-Atg5, LC3, Rab5, Rab7, LAMP1, and Pa Abs, respectively. (D) Lyn percent levels in different fractions were shown as means±SD. (E, F) MH-S cells were co-transfected with LAMP1-RFP, LC3-GFP, Ctrl or Lyn siRNA for 24 h, respectively. Cells were infected with PAO1 (MOI = 10, 1 h). CLSM imaging showed the localization of different puncta. Data are derived from 100 cells in each sample. (G) MH-S cells were infected with PAO1 (MOI = 10) for different times. Cell lysates were performed for immunoblotting with Pa and flagellin type b Abs, respectively. (H, I) Cells were infected with PAO1 as above. Immunostaining was used to detect pLyn and flagellin. The colocalization was detected by confocal microscope. The puncta were counted in each cell. Data are derived from 100 cells in each group. (J) MH-S cells were pretreated with Chloroquine (10 μM, 30 min). Cells were then infected with PAO1 as above for different times. Flagellin type b Abs was used to detect the digestion of bacterial proteins over time. (K) Schematic representation of Src kinase Lyn role in *P*. *aeruginosa*-induced autophagy. Upon Pa infection, free Lyn kinase in cytoplasm is activated and recruited to phagosome to facilitate autophagy derived phagocytosis. Data are shown as means+SD from three independent experiments. One-way ANOVA (Tukey’s post hoc); *, p<0.05. Scale bar = 5 μm.

To determine the critical role of Lyn in delivering bacterial components and in enabling intracellular traffic, a process required for autophagosome-lysosome fusion, LAMP1-RFP was transfected into macrophages to track lysosomes. We found that both LAMP1 and pLyn were colocalized with internalized Pa at 8 h post infection (Fig 7A and 7B). To biochemically characterize the role of Lyn and autophagy in Pa trafficking inside the cell, we isolated phagosomes from Pa-infected cells using a sucrose gradient method [16]. Horse radish peroxidase (HRP) was used to determine phagosome distribution and Pa Ab was used to show phagosome. We found that both Atg12-Atg5 and LC3 were colocalized with Lyn, and were shifted into early phagosome, late phagosome or phagolysosome fractions following infection (Fig 7C and 7D). Importantly, Pa bacterial proteins were detected using polyclonal Pa antibody in phagolysosome (45%; fractions 3, 4, 5), early phagosome (30%; fraction 6) and late phagosome (25%; fractions 7, 8, 9), while negligible in other fractions as determined by immunoblotting (Fig 7C). The presence of LAMP1 in fractions 9 and 10 indicates the autophagosome fusion with lysosome and a potential role of autophagy in bacterial delivery and clearance (Fig 7C). CLSM imaging showed that the colocalization of LC-3 and Lyn was drastically impaired by Lyn knockdown (Fig 7E and 7F). Importantly, proteolytic assay also showed that the degradation of the phagocytized bacteria (Figs 7G–7I, S7A), while the lysosome inhibitor chloroquine prevented the digestion of the invading bacteria (Figs 7J and S7B). Finally, we used another clinically significant Gram-negative bacterium, *K*. *pneumoniae* (Kp), to elucidate whether the autophagy related phagocytosis is a general phenomenon during bacterial infection. As expected, Kp infection also induced LC3 puncta around internalized bacteria, which was inhibited by Lyn deficiency or 3-MA (S7C–S7G Fig). To further dissect that autophagy-dependent innate immunity is generally important for immunity to *P*. *aeruginosa* or *K*. *pneumoniae* infections, additional strains (PAK, PA14, Kp, and Kp-Xen 39) were used to determine whether manipulation of autophagy impacts bacterial survival. Reduced bacterial killing was caused by autophagy inhibition or Lyn deficiency, indicating that Lyn-assisted autophagy is a general pathway for innate immunity against these microorganisms. These results collectively demonstrate that Lyn has an essential role in Gram-negative bacteria-induced autophagy, facilitating bacterial clearance. Fig 7K illustrates a model delineating the role of Lyn in Pa-induced autophagy related phagocytosis.

## Discussion

The current study demonstrates that TLR2/Lyn signaling plays a critical role in recruitment of autophagic components to bacteria-containing phagosome. We show that Lyn mediates the formation of the membrane isolation in the proximity of the Pa-containing phagosome in AM. Our data further reveal that Lyn transmits signaling for cytoskeleton protein-mediated intracellular transport following TLR2 activation, resulting in a merge between autophagosome and phagosome. This autophagosome-lysosome fusion helps eliminate the internalized Pa, leading to enhanced bacterial clearance. Further, blocking autophagy and/or depleting Lyn hamper the delivery of bacteria into lysosomes, thereby dampening bacterial eradication. Taken together, we characterize TLR2 and Lyn as new regulators of bacteria-induced autophagy and phagocytosis both *in vitro* and *in vivo*, providing new insight into the molecular mechanism of immune responses against acute bacterial infection.

Autophagy induced by intracellular bacterial infections has garnered increasing interest and has been shown to play crucial roles in host defense in immunological cells [23,24]. This autophagic role is relevant to the bacterial fates after phagocytes engulf some critical pathogenic bacteria, such as *M*. *tuberculosis*, *L*. *monocytogenes*, *S*. *enterica* and *S*. *flexneri* [24–27]. Previous reports have defined that macrophages with Atg7 deficiency could express enhanced scavenger receptor to phagocytize and retain *M*. *tuberculosis* [28]. However, whether autophagy can influence the uptake of extracellular bacteria, such as Pa or Kp, is largely unknown. A recent report showed that Pa infection enhanced autophagy [29], and that Atg7^-/-^ mice showed enhanced susceptibility to infection and impaired bacterial clearance [29]. However, there is no *in vivo* evidence to show reduced bacterial clearance associated with autophagy deficiency in Lyn^-/-^ mice [30,31]. Using mouse models, we show that autophagy related proteins contribute to xenophagy of extracellular bacteria Pa or Kp, thus affecting phagocytosis of host macrophages. Our data implicate that the role of autophagy varies with bacteria.

Previous studies indicate that PRRs may be activated in a PAMP-specific manner. The TLR5-mediated response was shown to be induced by flagellin, while LPS appears to link to the TLR4-mediated response [32,33]. LPS is one of the important structural components in the outer membrane of Gram-negative bacteria. CD14 facilitates the transport of LPS to the TLR4/MD-2 complex and regulates LPS recognition [34]. However, TLR2 and TLR4 recognize different bacterial cell wall components [35,36]. We show that Pa induces autophagy and that HKPa initiates host inflammatory responses through TLR2. We noted that LPS, a TLR4 ligand, also induced autophagy. Previous studies indicated that TLR2-mediated recruitment of LC3 to phagosomes may promote phagolysosome fusion [18]. Consistent with this observation, we showed that Lyn boosts phagocytosis of macrophages and facilitates subsequent intracellular trafficking processes.

Our previous studies showed that Lyn plays a role in Pa infection [16,17,31]. However, whether Lyn can regulate autophagy has remained elusive. Others show that blocking autophagy sensitizes prostate cancer cells towards Src family kinase inhibitors [37]. Interestingly, Lyn is reported to increase the survival of glioblastoma cells under starvation-induced autophagy [38]. Here, we identify a role of Lyn in initiating autophagy during Pa infection. Unlike LAP, typical double-membraned autophagosomes that contain bacteria are formed in our study, which is also specifically relevant to the activity of Lyn.

We also noted that blocking autophagy by 3-MA impaired phagocytosis. mTOR is a major negative regulatory axis of autophagy, thus inhibition of mTOR and its associated pathways can induce autophagy. We also found that rapamycin increases autophagy to elevate innate immune defense against Pa infection. These findings indicate that Lyn facilitates phagocytosis through autophagic mechanisms and serves as a link between phagosomes and autophagosomes. Thus, we delineate a novel mechanism by which Lyn mediates autophagy-related phagocytosis to benefit host against Pa.

We also demonstrate that Lyn’s activity is required for inhibiting Pa infection, as depletion of Lyn and/or Atg7 in macrophages resulted in increased Pa growth. Hence, the participation of Lyn and Atg7 suppressed Pa replication, whereas the absence of Lyn or Atg7 or other autophagic proteins (Atg5 and Beclin1) allowed for an uncontrolled bacterial growth. Murine macrophages lacking Lyn are defective in migration and in phagocytosis [17]. These observations suggest that the uptake of Pa may be impaired in Lyn-deficient macrophages. Previous studies suggest that intracellular growth of bacteria requires the halt of phagolysosome fusion [39]. This intracellular trafficking defect is observed in permissive macrophages, and most bacteria-containing vacuoles fuse with lysosomes to clear bacteria [40]. How autophagosome-lysosome fusion is modulated upon Pa infection is poorly understood. Our findings demonstrate that Lyn may modulate the delivery of bacteria to lysosomes, facilitating autophagosome maturation and subsequent bacterial clearance.

Phagosomes bind microtubules and actin filaments and migrate to interact with other compartments within the cell [41]. Our studies indicate that Lyn is involved in polymerization of F-actin, a cofilin-dependent process. Rab5, an early endosome marker, enables the recruitment of early endosomes to form nascent phagosomes and is also required for the recruitment of Rab7 (a late endosome marker), thus promoting phagolysosome fusion [42]. By isolating phagosomes to biochemically identify which phagosomal constituents interact with Lyn, we found that both Rab5 and Rab7 interact with Lyn, which may be downstream regulators following Lyn-mediated autophagy related phagocytosis. Thus Rab5 and Rab7 may be required for delivering bacterial components to lysosomes in Lyn-mediated Pa phagocytosis and autophagosome-lysosome fusion.

In summary, our studies represent autophagic benefit in host defense against Pa through a TLR2/Lyn axis. The contribution of Lyn to autophagy is found to facilitate the removal of intracellular bacteria within macrophages. Our results also demonstrate that TLR2 activates Lyn. Identification of both Lyn and TLR2 as novel regulatory factors in autophagy may implicate their therapeutic potential for control of Gram-negative bacterial infection.

## Materials and Methods

### Ethics statement

This study was carried out in strict accordance with the recommendations in the Guide for the Care and Use of Laboratory Animals of the National Institutes of Health. The protocols were approved by the Institutional Animal Care and Use Committee at the University of North Dakota, School of Medicine (Assurance Number: A3917-01). Dissections and injections were performed under anesthesia that was induced and maintained with ketamine hydrochloride and xylazine, and all efforts were made to minimize suffering.

### Mice

*atg7*-deficient (floxed *atg7* ^*-/-*^) mice were provided by Dr. Youwen He at Duke University and these mice were originally constructed by Masaaki Komatsu at Tokyo Metropolitan Institute of Medical Science [43]. To generate mice with a monocyte-specific knockout of Atg7, we have generated myeloid-specific conditional knockout (KO) mice by cross-breeding *atg7* ^*flox/flox*^ with *lyz* ^*2tm1(Cre)lfo>/j*^ mice (Jackson Laboratory, Bar Harbor, ME) [44]. The knockout was induced by injecting 0.1 mg of tamoxifen (Sigma, St Louis, MO) daily for 5 days [45]. *lyn* KO mice were provided by Dr. S. Li at University of Massachusetts. Age and sex matched wild-type (WT) mice (C57BL/6J) were used as controls [31].

### Cells

MH-S (ATCC CRL-2019) were cultured in RPMI 1640 medium supplemented with 10% fetal bovine serum (HyClone Laboratories, Logan, UT) and 100 U/ml of penicillin/streptomycin (P/S, Life Technologies, Rockville, MD) antibiotics in a 37°C incubator with 5% CO_2_. Mouse AM were isolated by bronchoalveolar lavage (BAL) [46]. In brief, trachea was cannulated with a 20-gauge catheter; 0.9 ml BAL buffer was instilled, flushed four times, and retrieved. A total of 3.0 ml BALF was retrieved from each mouse and cytospin slides prepared with 0.5 ml BALF were stained by HEMA-3 (Fisher, Rockford, IL) to enumerate leukocyte subtypes based on their cellular and nuclear morphological properties. After centrifugation at 2, 000 rpm, AM cells were resuspended and cultured in RPMI 1640 medium as above. MLE-12 (ATCC CRL-211) were cultured in HITES medium as above. Stable TLR2 expression HEK293 cells (ATCC CRL-157) using a pUNO-TLR2 plasmid were obtained from InvivoGen (San Diego, CA) and cultured in DMEM medium as above.

### Bacteria

*P*. *aeruginosa* wild-type (WT) strain PAO1 was a gift from Dr. Stephen Lory (Harvard Medical School, Boston, MA). The PAO1-GFP strain was obtained from Dr. Gerald Pier (Department of Microbiology and Molecular Genetics, Brigham and Women’s Hospital, Harvard Medical School). A Pa-cherry strain was obtained from Dr. John Singer of University of Maine (Orono, ME). Kp-GFP (ATCC 43816 serotype II) was kindly provided by Dr. Steven Clegg (University of Iowa Carver College of Medicine, Iowa City, IA).

### Infection experiments

Bacteria were grown overnight in lysogeny broth (LB) at 37°C with vigorous shaking. Next day, the bacteria were pelleted by centrifugation at 8, 000×g and resuspended in 10 ml of fresh LB broth, in which they were allowed to grow until the mid-logarithmic phase. Thereafter, the optical density (OD) at 600 nm was measured, and the density was adjusted to 0.25 OD (1 OD = 1×10^9^ cells/ml). Before infection, cells were washed once with PBS, and replaced with antibiotic-free medium immediately [47]. Cells were infected by Pa at multiplicity of infection (MOI) of 10:1 (bacteria-cells ratio) for 1 h. After infection, macrophages were washed 3 times with PBS, extracellular bacteria were removed by addition of 100 μg/ml polymyxin B and left in incubation for another 1 h. Phagocytosis was evaluated by counting colony forming unit (CFU) [16]. Bacterial clearance by AM cells was also determined using the CFU assay after 1 h infection, following with 1 h polymyxin B incubation and then 10 h culture in antibiotics free medium to gauge eradication of the internalized bacteria, indicating clearance of bacteria [48]. For *in vivo* experiments, 40 mg/kg ketamine was used for anesthesia. Mice were intranasal instilled with 1×10^7^ CFU of Pa (suspended in 25 μl PBS). Mice were sacrificed 24 h post infection. The lungs were collected for paraffin section. For survival experiments, mice (6/group) were infected as above and monitored till moribund.

### Cell transfection

Cells were transfected with LC3-RFP, LC3-GFP, Lyn-GFP, Rab5-RFP (dominant negative, DN) and Rab7-RFP (DN) in serum-free medium (Thermofisher Scientific), and achieved high efficiency using LipofectAmine 2000 reagent (Life Technologies, Grand Island, NY) following the manufacturer’s instructions. Similarly, we performed siRNA transfection assays [49].

### RT-PCR array

Real-time PCR profiling of mRNAs were conducted on a SYBR Green-based, RT^2^ *Profiler* PCR Array System (Cat# PAMM-084Z, Qiagen, Valencia, CA). The array includes 84 key genes that encode components of the molecular machinery and key regulators modulating autophagy in response to both extracellular and intracellular signals. Expression of mRNAs was detected by QuantiTect SYBR Green RT–PCR Kit (Qiagen, Valencia, CA). The separate well 2-ΔΔCt cycle threshold method was used to determine relative quantitative levels of individual mRNA, and these were expressed as the fold difference to GAPDH, respectively.

### Immunoblotting analysis

Rabbit polyclonal antibodies against, Atg7, Atg12-Atg5, TLR2, Lyn, pcofilin-1, cofilin-1, flotillin-1, actin and mouse polyclonal antibodies against LC3, GAPDH were obtained from Santa Cruz Biotechnology (Santa Cruz, CA). Rabbit monoclonal antibody against phosphor-Lyn (Tyr297) was obtained from Cell Signaling Technology (Danvers, MA). Rabbit monoclonal antibody against flagellin type b and Pa were kindly provided by Dr. Gerald Pier of Harvard Medical School. The samples from cells were lysed and quantified. The lysates were boiled for 10 min, and added with protease inhibitor cocktail (Roche Diagnostics, Indianapolis, IN). The supernatants were collected and 20 μg of each sample were loaded onto 12% SDS-polyacrylamide gel electrophoresis (PAGE) and electrophoresed for protein resolution. The proteins were then transferred to polyvinylidine difluoride membranes (Thermofisher, Rockford, IL) and blocked for 1 h at room temperature using 5% non-fat milk blocking buffer. Membranes were incubated for 2 h at room temperature with appropriate first antibodies diluted at 1:1000 in 5% bovine serum albumin (BSA) immunoblotting antibody buffer. After washing (three times, 10 min once) with washing solution, the membranes were incubated for 60 min at room-temperature with horseradish peroxidase-conjugated secondary antibody (Rockland Immunochemicals, Gilbertsville, PA) diluted 1:3000. Signals were visualized using an enhanced chemiluminescence detection kit (SuperSignal West Pico, Pierce) [46]. Co-immunoprecipitation (co-IP) is performed using Protein A/G-argarose beads and then mix with the related antibody. The target proteins were detected using immunoblotting as above.

### Confocal microscopy and indirect immunofluorescence staining

Cells were grown either on coverslips in a 24-well plate or in glass-bottomed dishes (MatTek, Ashland, MA). Rabbit monoclonal antibody against Ly6G was bought from Abcam (Cambridge, MA). For immunostaining, the cells or slide tissues were fixed in 3.7% paraformaldehyde, permeabilized with 0.2% Triton X-100 in PBS and incubated with blocking buffer containing 2% BSA for 30 min. Samples were incubated with primary antibodies at 1/500 dilution in blocking buffer for 1 h and washed three times. After incubation with appropriate fluorophore-conjugated secondary antibodies, the cover slips were mounted on slides with Vectashield mounting medium. The images were captured using an LSM 510 Meta confocal laser scanning microscope (CLSM, Carl Zeiss Micro Imaging, Dublin, CA), and processed using the software provided by the manufacturer [50].

### GST-Lyn pull-down assay

GST-Lyn constructs with different functional domains were originally obtained from Dr. O. Miura (Tokyo Medical and Dental University, Tokyo, Japan) [22] and transformed into BL21-DE3 strain of *E*. *coli*. The GST-Lyn fragments were extracted using immobilized glutathione columns following the manufacturer’s instructions [16]. Equal whole cell lysates were incubated with GST-Lyn peptide for protein interactions. The pull-down products were analyzed by immunoblotting with specific Abs.

### Phagosome isolation

Cell lysates were processed for phagosome isolation, as previously described [51]. Briefly, the cell homogenates were separated by sucrose gradient density centrifugation ranging from 90 to 5% of sucrose cushion. The phagosomes were heavy and separated at 65% density, whereas lysosomes were found at the interface of 35 and 5% densities. Fractions (1.1 ml) were collected from top (total 10 fractions) corresponding to the density gradient.

### Transmission electron microscopy (TEM)

TEM was employed for identifying autophagosomes using modified Karnovsky’s fixative [52]. Images were taken and processed according to our previous reports [30].

### Statistical analysis

All experiments were performed in triplicate and repeated at least three times. Data are presented as changes compared with the controls from the three independent experiments. Results are shown as means±SD. One-way ANOVA (Tukey’s post hoc) was used for statistical analysis. Differences were accepted as significant at p<0.05.

## Supporting Information

S1 Fig mRNA microarray expressing profiling analysis. (A) MH-S cells were infected with PAO1 (MOI = 10, 1 h). Microarray analysis of mRNA expression in Pa-infected cells versus medium only controls. Genes with >4 fold change among subsets are highlighted in Red. (B) Heat map representation of differentially expressed mRNAs in cells infected with Pa compared with normal cells. Red indicates up-regulated mRNAs, and green indicates down-regulated mRNAs.
(TIF)

S2 Fig Lyn affects autophagy upon Pa infection both *in vitro* and *in vivo*. (A) MH-S cells were transfected with Ctrl or Atg7 siRNA at 10 nM for 24 h, respectively. Mouse primary AM from WT or Atg7^-/-^ mice were collected by bronchoalveolar lavage (BAL). Cell lysates were performed for immunoblotting to detect the expression of Atg7. GAPDH was used as loading control in the whole manuscript. (B, C) Cells were infected with PAO1 (MOI = 10). Cells viability and phagocytosis and clearance were tested using MTT assay or CFU assay, respectively. (D, E) Normal MH-S cells were pretreated with autophagy inducer (rapamycin, 500 nM, 12 h) or inhibitor (3-MA, 5 mM, 3 h), then infected with PAO1, MTT assays and CFU assays were performed as above. (F) MLE-12 cells were co-transfected with LC3-RFP and Ctrl or Lyn siRNA for 24 h, respectively. Cells were pretreated with rapamycin (500 nM, 12 h) or 3-MA (5 mM, 3 h), and then infected with PAO1 (MOI = 10, 1 h). CLSM images show significant LC3 puncta upon Pa infection. Arrows indicate LC3 puncta. Scale bar = 5 μm. (G) Mice infected with PAO1 for 24 h in Fig 1M were collected and performed for CFU assay. (H, I) Lungs from above were performed for histological analysis (inset showing the typical tissue injury and inflammatory influx); and immunostaining were performed to detect the expression of Ly6G. Scale bar = 50 μm. Data are representative and shown as means+SD from three independent experiments. One-way ANOVA (Tukey’s post hoc); *, p<0.05.
(TIF)

S3 Fig Densitometry for protein quantitative analysis. (A) Quantification of pLyn and LC3-II level in Fig 2C is shown. (B) Quantification of LC3-II level in Fig 2F is shown. (C) Quantification of LC3-II level in Fig 2G is shown. (D) MH-S cells were treated with Zymosan (10 μg/ml, 1 h). Cells viability were tested by MTT assay. (E) Quantification of LC3-II expression in Fig 2J is shown. (F) MH-S cells were co-transfected with Lyn-GFP and LC3-RFP for 24 h. Cells were infected with Strep Pyogenes (Sp, MOI = 10, 1 h). The colocalization of Lyn and LC3 was found by CLSM imaging. Scale bar = 5 μm. (G) MH-S cells were transfected with Ctrl, Rubicon or ULK1 siRNA, respectively, and performed for Immunoblotting. (H) Quantification of pLyn and LC3-II level in Fig 2O is shown. (I) MH-S cells were pretreated with Chloroquine (40 μM, 6 h). Cells were infected with PAO1 (MOI = 10, 1 h). Cell lysates were performed for immunoblotting. (J) Quantification of pLyn and LC3-II level in S3H Fig is shown. Data are representative and shown as means+SD from three independent experiments. One-way ANOVA (Tukey’s post hoc); *, p<0.05.
(TIF)

S4 Fig Lyn activations is required for infection-induced autophagy. (A) GST-Lyn-A and GST-Lyn 1–230 were used to detect the *in vitro* association with or without Pa (PAO1, MOI = 10, 1 h) infection. (B) Quantification of Atg12-Atg5, LC3 and Pa protein levels in S4A Fig is shown. (C) MLE-12 cells and Primary AMs were infected with Pa as above, and then lysed for pulldown assay. GST-Lyn 1–230 containing both SH3 and SH2 domains shows association with Atg5-Atg12, LC3 and Pa in AMs. (D) Quantification of Atg12-Atg5, LC3 and Pa protein levels in Fig 3G and S4C Fig is shown. (E) Quantification of pLyn and LC3-II level in Fig 3J is shown. (F) MH-S cells were pretreated with PP2 (5 nM, 30 min). Cells were then infected with PAO1 as above and then lysed for immunoblotting to detect pLyn, Lyn and LC3. (G) Quantification of pLyn and LC3-II level in S4D Fig is shown. Data are representative and shown as means+SD from three independent experiments. One-way ANOVA (Tukey’s post hoc); *, p<0.05; **, p<0.01.
(TIF)

S5 Fig TLR2 is involved in Lyn-mediated autophagy. (A) Quantification of pLyn and LC3-II level in Fig 4A is shown. (B) Quantification of pLyn and LC3-II level in Fig 4I is shown. (C) Quantification of pLyn and LC3-II level in Fig 4J is shown. (D) MH-S cells were pretreated with Pam_3_CSK_4_ (300 ng/ml), and infected with PAO1 (1 h). Cells viability was determined using MTT assay. (E) MH-S cells were transfected with LC3-RFP for 24 h and then treated as above. CLSM imaging was used to detect LC3 puncta. (F) Quantification of TLR2, pLyn and LC3-II level in Fig 4N is shown. Data are representative and shown as means+SD from three independent experiments. One-way ANOVA (Tukey’s post hoc); *, p<0.05. Scale bar = 5 μm.
(TIF)

S6 Fig Lyn affects canonical phagocytosis through Rab5 and Rab7. (A) MH-S cells were infected with PAO1 (MOI = 10, 1 h). Cells were lysed for Co-IP to detect the interaction of Lyn with Rab5 and Rab7. (B, C) MH-S cells were co-transfected with Rab7-RFP and Ctrl or Lyn siRNA for 24 h. The cells were then infected with PAO1-GFP (MOI = 10, 8 h). CLSM imaging was used to detect related pores and the number of puncta in each cell was shown. Data are derived from 100 cells in each group. Scale bar = 5 μm. (D, E) MH-S cells were transfected with Rab5-RFP or Rab5-DN-RFP plasmid for 24 h. Then the cells were infected with PAO1-GFP. Colocalization between Rab5 and Pa was monitored. Arrows indicate the colocalized puncta and quantification was performed over time. Data are derived from 100 cells in each group. Scale bar = 25 μm. (F, G) MH-S cells were transfected with Rab7-RFP or Rab7-DN-RFP plasmid for 24 h and infected with PAO1-GFP. The internalized bacteria in each cell were counted in the lasting 12 h. Data are derived from 100 cells in each group. Scale bar = 25 μm. (H, I) MH-S cells were infected with PAO1 (8 h) and were homogenized. Cell lysates were immunoblotted with antibodies against phosphorylated Cofilin-1 (pCofilin-1), Cofilin-1, Flotillin-1, and Actin. The protein levels of pCofilin-1 and Flotillin-1 were quantified. (J) Whole cell lysates were immunoprecipitated (IP) with beads coated with Lyn antibody and immunoblotted with Cofilin-1 and Flotillin-1 antibody. Data are representative and shown as means+SD from three independent experiments. One-way ANOVA (Tukey’s post hoc); *, p<0.05.
(TIF)

S7 Fig *K*. *pneumoniae* also induces autophagy in macrophages. (A) Quantification of Pa and Flagellin level in Fig 7G is shown. (B) Quantification of Flagellin level in Fig 7J is shown. (C) MH-S cells were transfected with Ctrl or Lyn siRNA for 24 h. Cells were pretreated with rapamycin (500 nM, 12 h) or 3-MA (5 mM, 3 h), then infected with Kp (MOI = 10, 1 h). MTT assays were performed to test cell viability. (D) Cell lysates were collected and immunoblotting of Atg12-Atg5 and LC3 was performed. (E) Quantification of Atg12-Atg5 and LC3-II level in S7D Fig is shown. (F, G) MH-S cells were co transfected with LC3-RFP plasmid with Ctrl or Lyn siRNA, respectively. The cells were then infected with Kp-GFP (MOI = 10, 1 h). Colocalization between LC3 and Kp was monitored. Arrows indicate the colocalized puncta. Quantification of LC3 puncta in each cell was performed. The percentage of LC3^+^/Kp^+^ events (cell with colocalized puncta of LC3-RFP and Kp-GFP) is shown. Data are derived from 100 cells in each sample. Scale bar = 5 μm. (H) MH-S cells were pretreated with rapamycin (500 nM, 12 h) or 3-MA (5 mM, 3 h), or transfected with Ctrl or Lyn siRNA, respectively. Cells were then infected with bacteria (MOI = 10), respectively. CFU assays were performed as above to show phagocytosis or clearance upon bacterial infection. Data are representative and shown as means+SD from three independent experiments. One-way ANOVA (Tukey’s post hoc); *, p<0.05.
(TIF)

S1 Table Micro-array data of autophagy related genes expression. (DOCX)
