# Supplementary figures and images for: Lyn Delivers Bacteria to Lysosomes for Eradication through TLR2-Initiated Autophagy Related Phagocytosis
Source: PLoS Pathog. 2016 Jan 6;12(1):e1005363. doi: 10.1371/journal.ppat.1005363 (PMC4703367; doi:10.1371/journal.ppat.1005363)

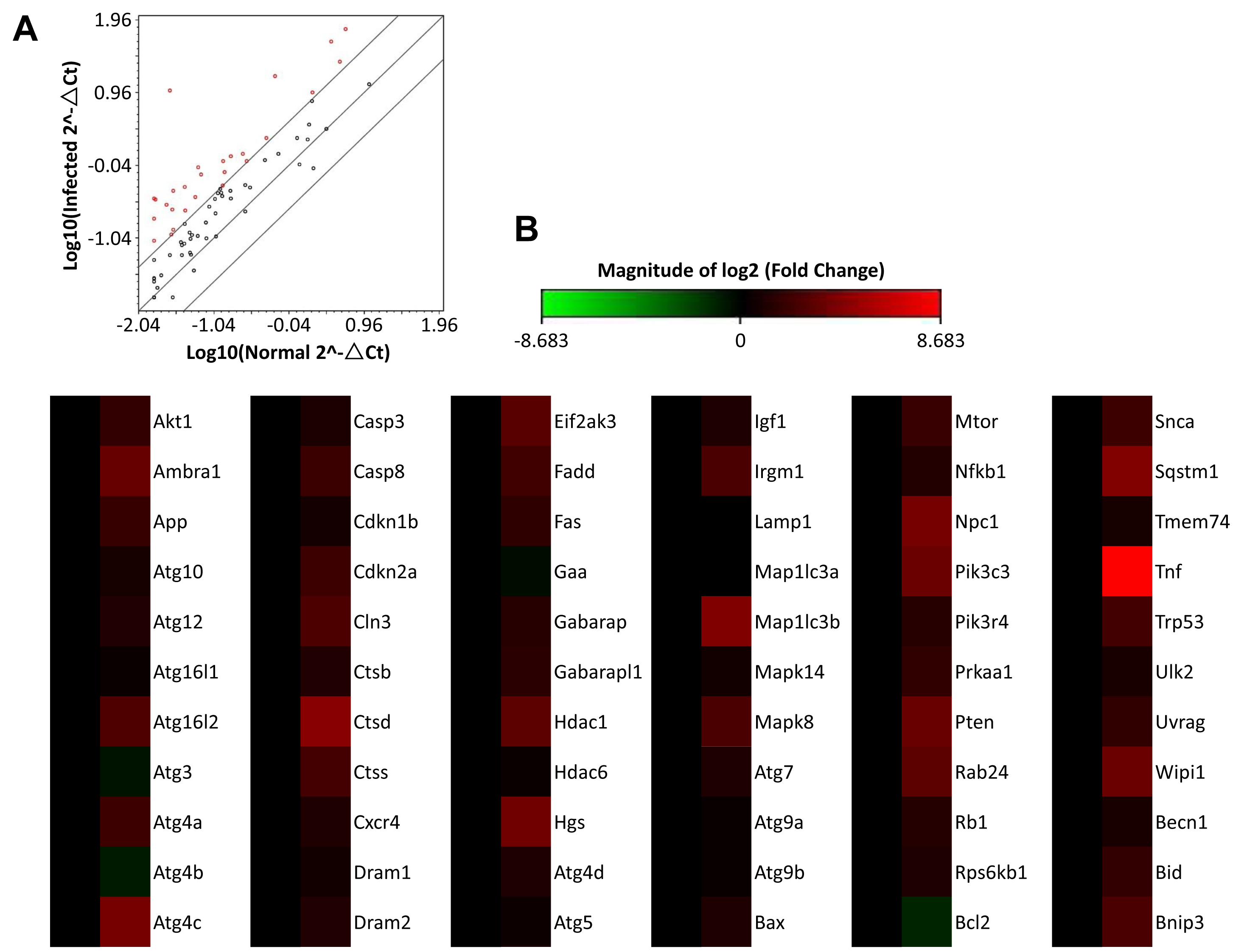

Supplement: S1 Fig — (A) MH-S cells were infected with PAO1 (MOI = 10, 1 h). Microarray analysis of mRNA expression in Pa-infected cells versus medium only controls. Genes with >4 fold change among subsets are highlighted in Red. (B) Heat map representation of differentially expressed mRNAs in cells infected with Pa compared with normal cells. Red indicates up-regulated mRNAs, and green indicates down-regulated mRNAs. (TIF) [file ppat.1005363.s001.tif]

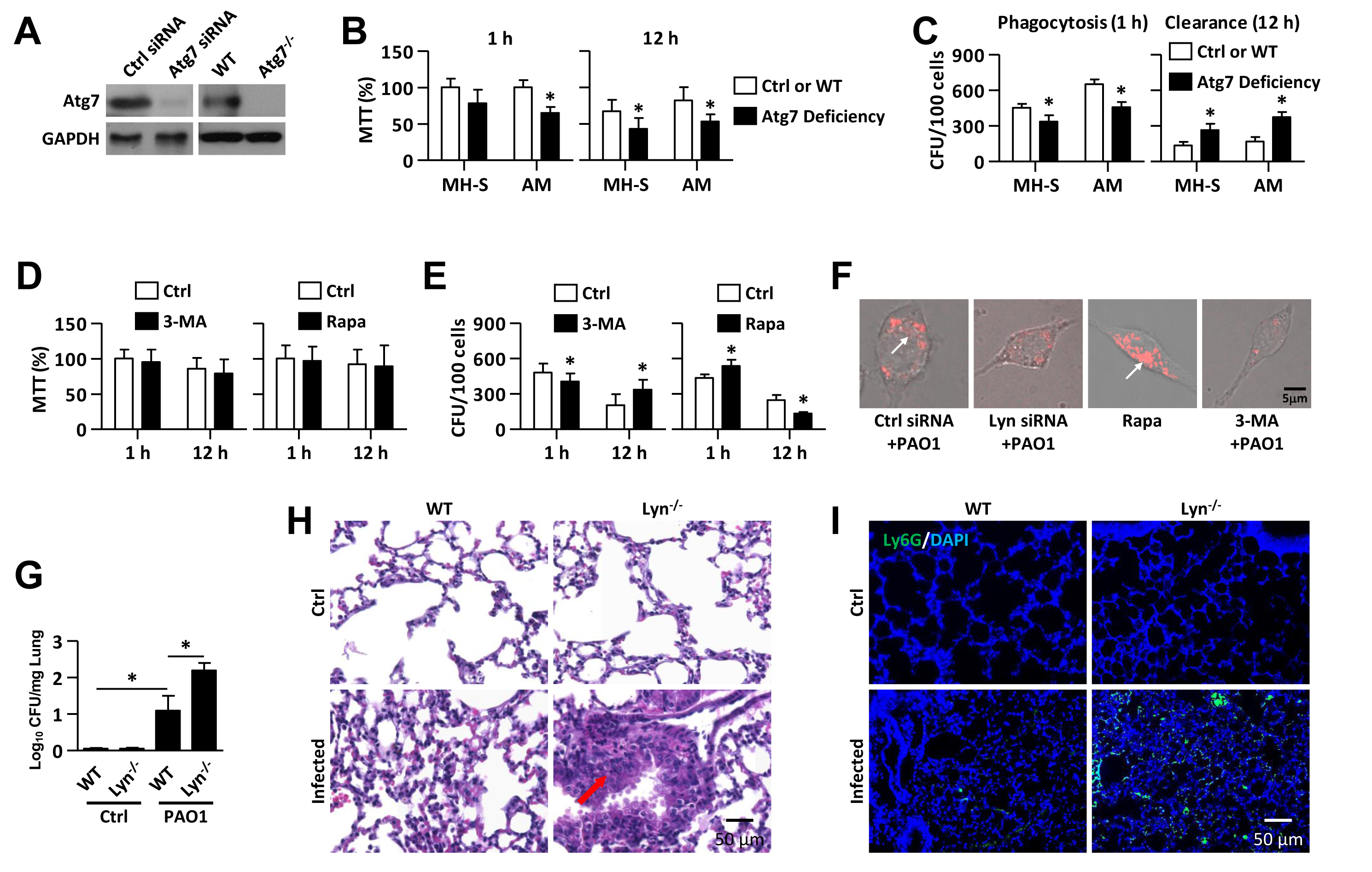

Supplement: S2 Fig — (A) MH-S cells were transfected with Ctrl or Atg7 siRNA at 10 nM for 24 h, respectively. Mouse primary AM from WT or Atg7-/- mice were collected by bronchoalveolar lavage (BAL). Cell lysates were performed for immunoblotting to detect the expression of Atg7. GAPDH was used as loading control in the whole manuscript. (B, C) Cells were infected with PAO1 (MOI = 10). Cells viability and phagocytosis and clearance were tested using MTT assay or CFU assay, respectively. (D, E) Normal MH-S cells were pretreated with autophagy inducer (rapamycin, 500 nM, 12 h) or inhibitor (3-MA, 5 mM, 3 h), then infected with PAO1, MTT assays and CFU assays were performed as above. (F) MLE-12 cells were co-transfected with LC3-RFP and Ctrl or Lyn siRNA for 24 h, respectively. Cells were pretreated with rapamycin (500 nM, 12 h) or 3-MA (5 mM, 3 h), and then infected with PAO1 (MOI = 10, 1 h). CLSM images show significant LC3 puncta upon Pa infection. Arrows indicate LC3 puncta. Scale bar = 5 μm. (G) Mice infected with PAO1 for 24 h in Fig 1M were collected and performed for CFU assay. (H, I) Lungs from above were performed for histological analysis (inset showing the typical tissue injury and inflammatory influx); and immunostaining were performed to detect the expression of Ly6G. Scale bar = 50 μm. Data are representative and shown as means+SD from three independent experiments. One-way ANOVA (Tukey’s post hoc); *, p<0.05. (TIF) [file ppat.1005363.s002.tif]

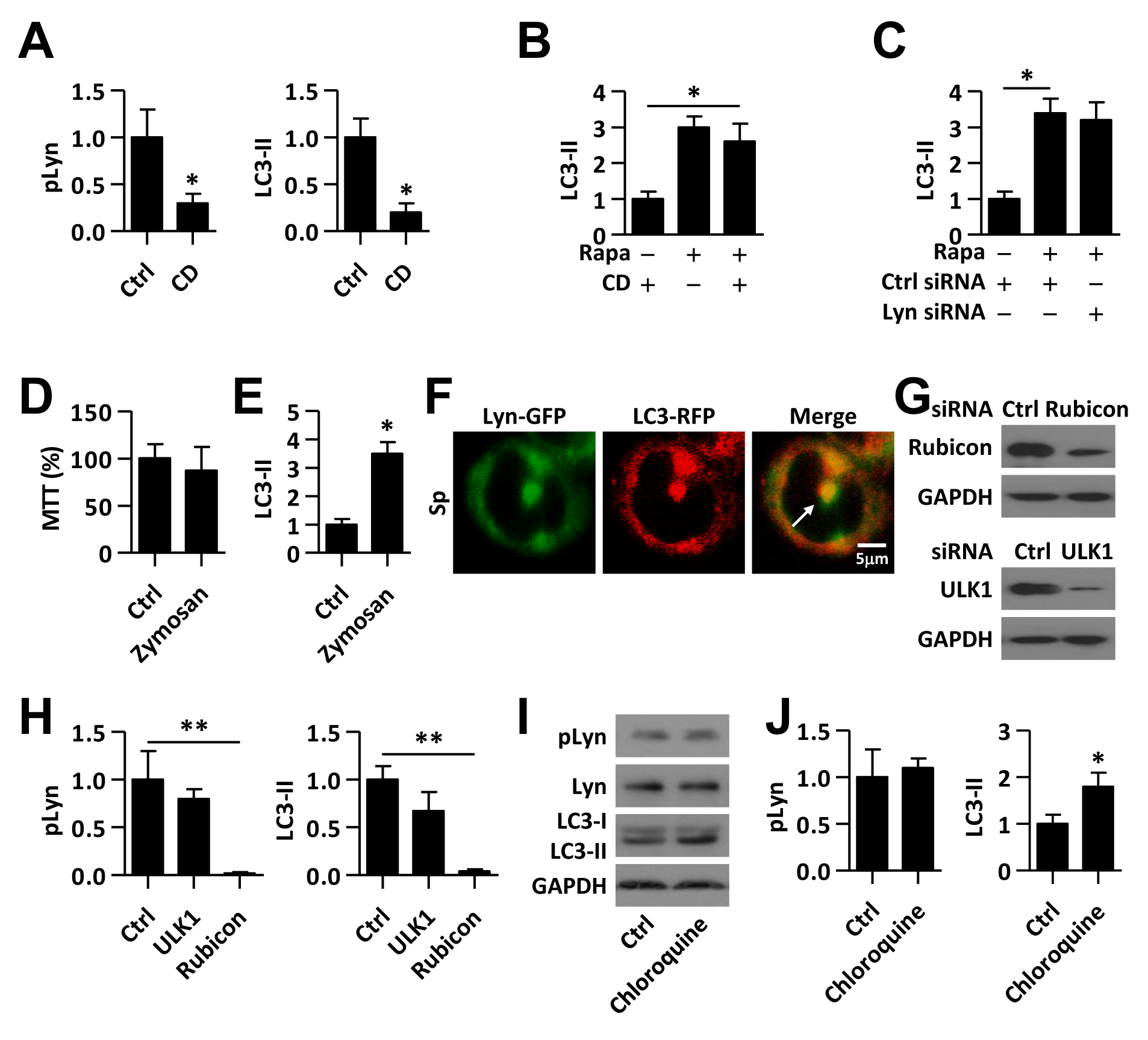

Supplement: S3 Fig — (A) Quantification of pLyn and LC3-II level in Fig 2C is shown. (B) Quantification of LC3-II level in Fig 2F is shown. (C) Quantification of LC3-II level in Fig 2G is shown. (D) MH-S cells were treated with Zymosan (10 μg/ml, 1 h). Cells viability were tested by MTT assay. (E) Quantification of LC3-II expression in Fig 2J is shown. (F) MH-S cells were co-transfected with Lyn-GFP and LC3-RFP for 24 h. Cells were infected with Strep Pyogenes (Sp, MOI = 10, 1 h). The colocalization of Lyn and LC3 was found by CLSM imaging. Scale bar = 5 μm. (G) MH-S cells were transfected with Ctrl, Rubicon or ULK1 siRNA, respectively, and performed for Immunoblotting. (H) Quantification of pLyn and LC3-II level in Fig 2O is shown. (I) MH-S cells were pretreated with Chloroquine (40 μM, 6 h). Cells were infected with PAO1 (MOI = 10, 1 h). Cell lysates were performed for immunoblotting. (J) Quantification of pLyn and LC3-II level in S3H Fig is shown. Data are representative and shown as means+SD from three independent experiments. One-way ANOVA (Tukey’s post hoc); *, p<0.05. (TIF) [file ppat.1005363.s003.tif]

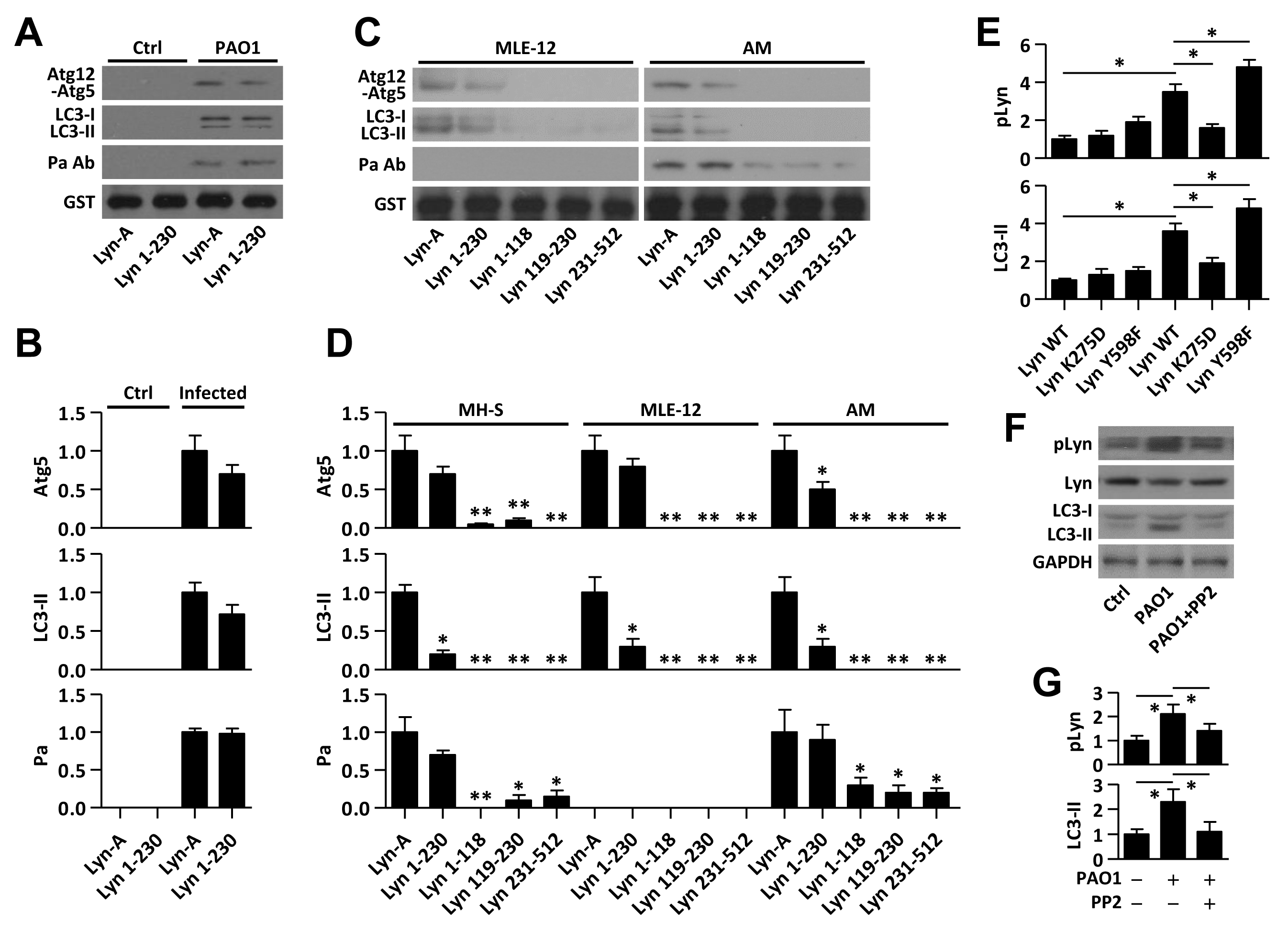

Supplement: S4 Fig — (A) GST-Lyn-A and GST-Lyn 1–230 were used to detect the in vitro association with or without Pa (PAO1, MOI = 10, 1 h) infection. (B) Quantification of Atg12-Atg5, LC3 and Pa protein levels in S4A Fig is shown. (C) MLE-12 cells and Primary AMs were infected with Pa as above, and then lysed for pulldown assay. GST-Lyn 1–230 containing both SH3 and SH2 domains shows association with Atg5-Atg12, LC3 and Pa in AMs. (D) Quantification of Atg12-Atg5, LC3 and Pa protein levels in Fig 3G and S4C Fig is shown. (E) Quantification of pLyn and LC3-II level in Fig 3J is shown. (F) MH-S cells were pretreated with PP2 (5 nM, 30 min). Cells were then infected with PAO1 as above and then lysed for immunoblotting to detect pLyn, Lyn and LC3. (G) Quantification of pLyn and LC3-II level in S4D Fig is shown. Data are representative and shown as means+SD from three independent experiments. One-way ANOVA (Tukey’s post hoc); *, p<0.05; **, p<0.01. (TIF) [file ppat.1005363.s004.tif]

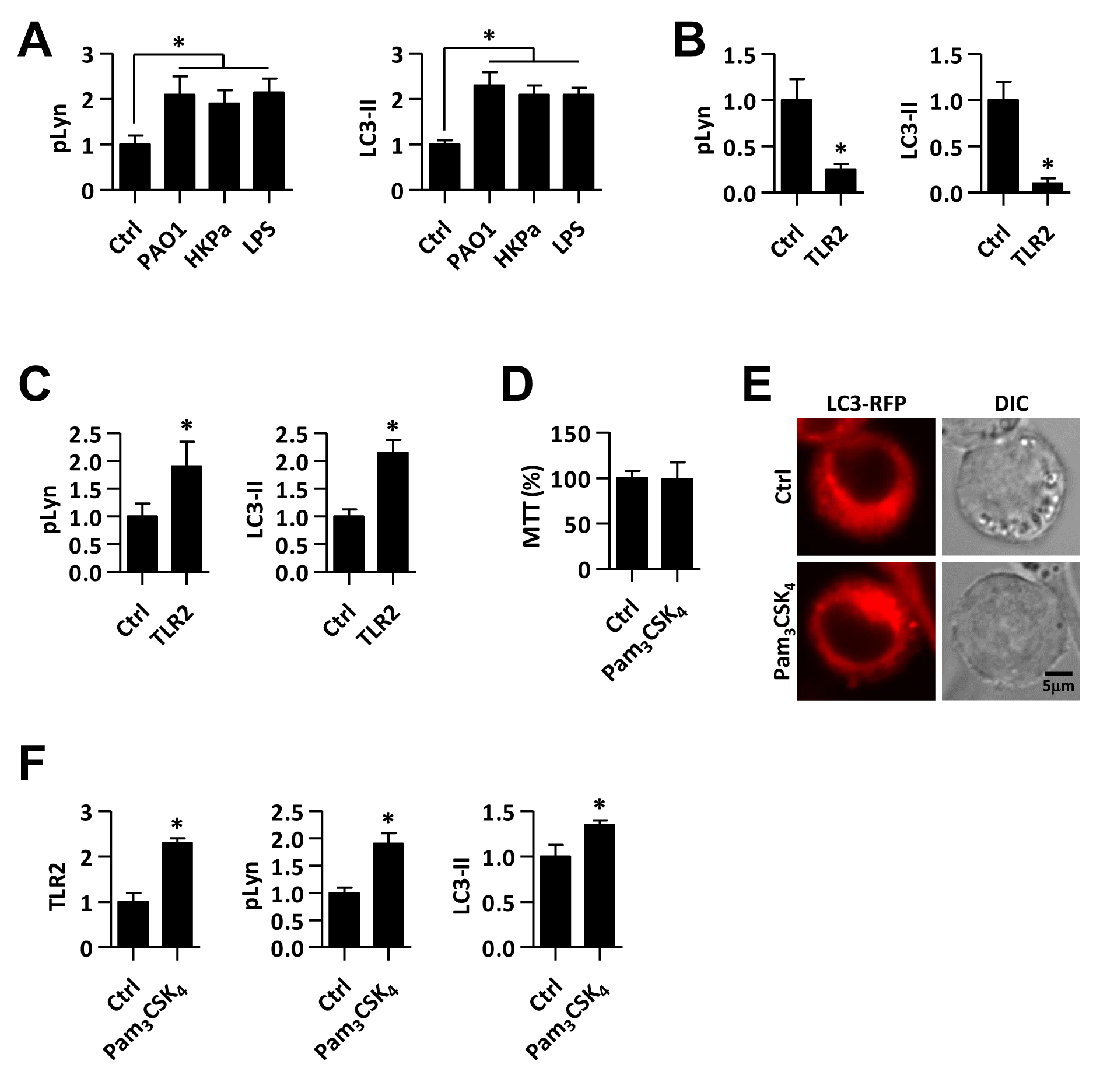

Supplement: S5 Fig — (A) Quantification of pLyn and LC3-II level in Fig 4A is shown. (B) Quantification of pLyn and LC3-II level in Fig 4I is shown. (C) Quantification of pLyn and LC3-II level in Fig 4J is shown. (D) MH-S cells were pretreated with Pam3CSK4 (300 ng/ml), and infected with PAO1 (1 h). Cells viability was determined using MTT assay. (E) MH-S cells were transfected with LC3-RFP for 24 h and then treated as above. CLSM imaging was used to detect LC3 puncta. (F) Quantification of TLR2, pLyn and LC3-II level in Fig 4N is shown. Data are representative and shown as means+SD from three independent experiments. One-way ANOVA (Tukey’s post hoc); *, p<0.05. Scale bar = 5 μm. (TIF) [file ppat.1005363.s005.tif]

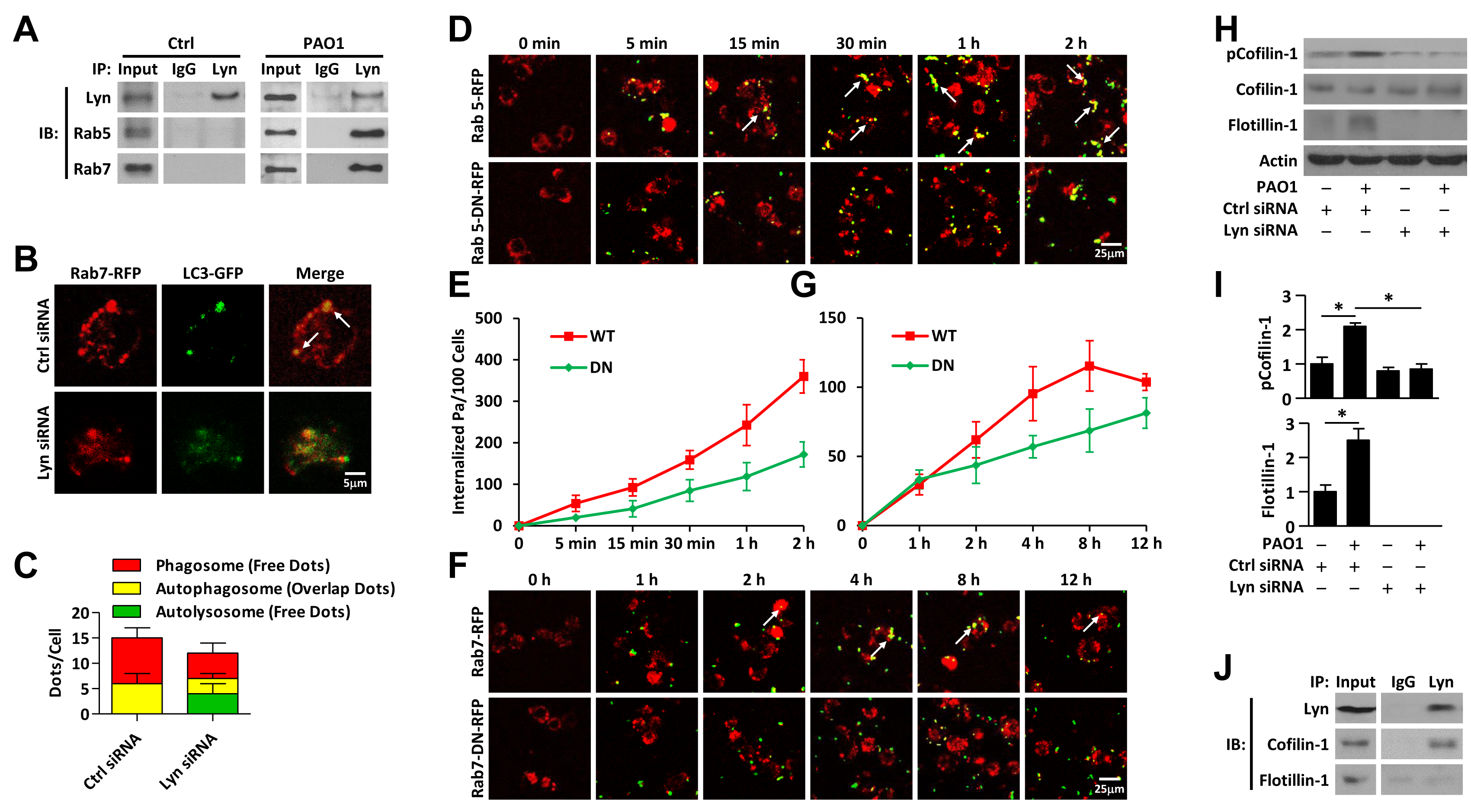

Supplement: S6 Fig — (A) MH-S cells were infected with PAO1 (MOI = 10, 1 h). Cells were lysed for Co-IP to detect the interaction of Lyn with Rab5 and Rab7. (B, C) MH-S cells were co-transfected with Rab7-RFP and Ctrl or Lyn siRNA for 24 h. The cells were then infected with PAO1-GFP (MOI = 10, 8 h). CLSM imaging was used to detect related pores and the number of puncta in each cell was shown. Data are derived from 100 cells in each group. Scale bar = 5 μm. (D, E) MH-S cells were transfected with Rab5-RFP or Rab5-DN-RFP plasmid for 24 h. Then the cells were infected with PAO1-GFP. Colocalization between Rab5 and Pa was monitored. Arrows indicate the colocalized puncta and quantification was performed over time. Data are derived from 100 cells in each group. Scale bar = 25 μm. (F, G) MH-S cells were transfected with Rab7-RFP or Rab7-DN-RFP plasmid for 24 h and infected with PAO1-GFP. The internalized bacteria in each cell were counted in the lasting 12 h. Data are derived from 100 cells in each group. Scale bar = 25 μm. (H, I) MH-S cells were infected with PAO1 (8 h) and were homogenized. Cell lysates were immunoblotted with antibodies against phosphorylated Cofilin-1 (pCofilin-1), Cofilin-1, Flotillin-1, and Actin. The protein levels of pCofilin-1 and Flotillin-1 were quantified. (J) Whole cell lysates were immunoprecipitated (IP) with beads coated with Lyn antibody and immunoblotted with Cofilin-1 and Flotillin-1 antibody. Data are representative and shown as means+SD from three independent experiments. One-way ANOVA (Tukey’s post hoc); *, p<0.05. (TIF) [file ppat.1005363.s006.tif]

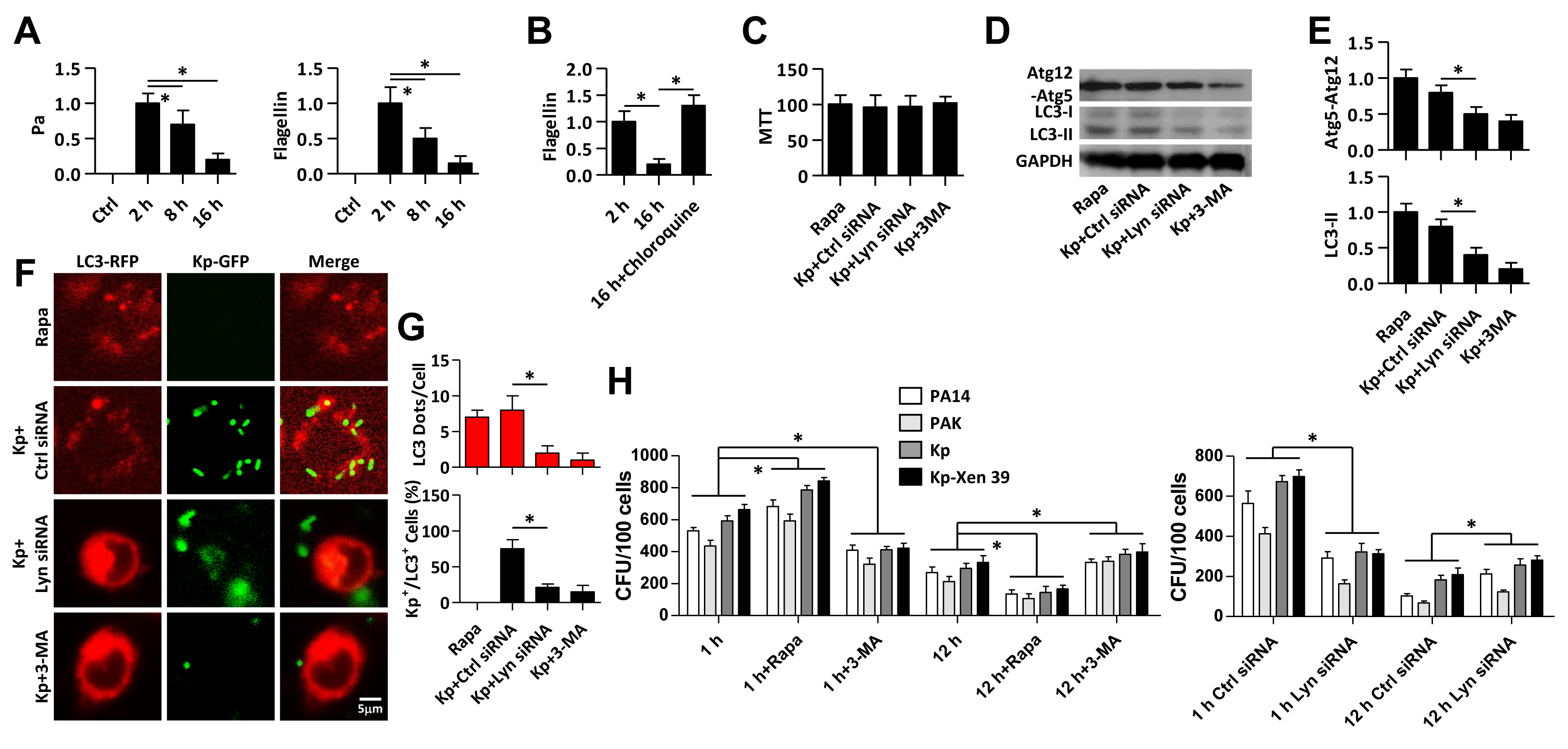

Supplement: S7 Fig — (A) Quantification of Pa and Flagellin level in Fig 7G is shown. (B) Quantification of Flagellin level in Fig 7J is shown. (C) MH-S cells were transfected with Ctrl or Lyn siRNA for 24 h. Cells were pretreated with rapamycin (500 nM, 12 h) or 3-MA (5 mM, 3 h), then infected with Kp (MOI = 10, 1 h). MTT assays were performed to test cell viability. (D) Cell lysates were collected and immunoblotting of Atg12-Atg5 and LC3 was performed. (E) Quantification of Atg12-Atg5 and LC3-II level in S7D Fig is shown. (F, G) MH-S cells were co transfected with LC3-RFP plasmid with Ctrl or Lyn siRNA, respectively. The cells were then infected with Kp-GFP (MOI = 10, 1 h). Colocalization between LC3 and Kp was monitored. Arrows indicate the colocalized puncta. Quantification of LC3 puncta in each cell was performed. The percentage of LC3+/Kp+ events (cell with colocalized puncta of LC3-RFP and Kp-GFP) is shown. Data are derived from 100 cells in each sample. Scale bar = 5 μm. (H) MH-S cells were pretreated with rapamycin (500 nM, 12 h) or 3-MA (5 mM, 3 h), or transfected with Ctrl or Lyn siRNA, respectively. Cells were then infected with bacteria (MOI = 10), respectively. CFU assays were performed as above to show phagocytosis or clearance upon bacterial infection. Data are representative and shown as means+SD from three independent experiments. One-way ANOVA (Tukey’s post hoc); *, p<0.05. (TIF) [file ppat.1005363.s007.tif]
