# Supplementary material for: Lyn Delivers Bacteria to Lysosomes for Eradication through TLR2-Initiated Autophagy Related Phagocytosis
Source: PLoS Pathog. 2016 Jan 6;12(1):e1005363. doi: 10.1371/journal.ppat.1005363 (PMC4703367; doi:10.1371/journal.ppat.1005363)
Supplement: S1 Table — (DOCX) [file ppat.1005363.s008.docx]

**Supplementary Table 1. Micro-array data of autophagy related genes expression**

| **Refseq** | **Symbol** | **Description** | **Fold Change** |
| --- | --- | --- | --- |
| NM_009652 | Akt1 | Thymoma viral proto-oncogene 1 | 3.3302 |
| NM_172669 | Ambra1 | Autophagy/beclin 1 regulator 1 | 11.131 |
| NM_007471 | App | Amyloid beta (A4) precursor protein | 3.6272 |
| NM_009713 | Arsa | Arylsulfatase A | 1.7154 |
| NM_025770 | Atg10 | Autophagy-related 10 (yeast) | 2.1355 |
| NM_026217 | Atg12 | Autophagy-related 12 (yeast) | 1.2442 |
| NM_029846 | Atg16l1 | Autophagy-related 16-like 1 (yeast) | 6.3841 |
| NM_001111111 | Atg16l2 | Autophagy related 16 like 2 (S. cerevisiae) | -1.5169 |
| NM_026402 | Atg3 | Autophagy-related 3 (yeast) | 4.1983 |
| NM_174875 | Atg4a | Autophagy-related 4A (yeast) | -1.8616 |
| NM_174874 | Atg4b | Autophagy-related 4B (yeast) | 15.4356 |
| NM_175029 | Atg4c | Autophagy-related 4C (yeast) | 2.076 |
| NM_153583 | Atg4d | Autophagy-related 4D (yeast) | 1.2967 |
| NM_053069 | Atg5 | Autophagy-related 5 (yeast) | 2.0518 |
| NM_028835 | Atg7 | Autophagy-related 7 (yeast) | 1.1893 |
| NM_001003917 | Atg9a | Autophagy-related 9A (yeast) | 1.1809 |
| NM_001002897 | Atg9b | ATG9 autophagy related 9 homolog B (S. cerevisiae) | -1.0539 |
| NM_007522 | Bad | BCL2-associated agonist of cell death | -1.1243 |
| NM_007523 | Bak1 | BCL2-antagonist/killer 1 | 2.0763 |
| NM_007527 | Bax | Bcl2-associated X protein | -2.3173 |
| NM_009741 | Bcl2 | B-cell leukemia/lymphoma 2 | -1.0539 |
| NM_009743 | Bcl2l1 | Bcl2-like 1 | 1.7462 |
| NM_019584 | Becn1 | Beclin 1, autophagy related | 3.329 |
| NM_007544 | Bid | BH3 interacting domain death agonist | 5.7534 |
| NM_009760 | Bnip3 | BCL2/adenovirus E1B interacting protein 3 | 1.8309 |
| NM_009810 | Casp3 | Caspase 3 | 3.8478 |
| NM_009812 | Casp8 | Caspase 8 | 1.5704 |
| NM_009875 | Cdkn1b | Cyclin-dependent kinase inhibitor 1B | 4.1463 |
| NM_009877 | Cdkn2a | Cyclin-dependent kinase inhibitor 2A | 5.8863 |
| NM_009907 | Cln3 | Ceroid lipofuscinosis, neuronal 3, juvenile (Batten, Spielmeyer-Vogt disease) | 2.145 |
| NM_007798 | Ctsb | Cathepsin B | 25.8394 |
| NM_021281 | Ctss | Cathepsin S | 4.8786 |
| NM_009911 | Cxcr4 | Chemokine (C-X-C motif) receptor 4 | 2.0715 |
| NM_029653 | Dapk1 | Death associated protein kinase 1 | -1.0539 |
| NM_027878 | Dram1 | DNA-damage regulated autophagy modulator 1 | 1.5369 |
| NM_010121 | Eif2ak3 | Eukaryotic translation initiation factor 2 alpha kinase 3 | 2.0832 |
| NM_001005331 | Eif4g1 | Eukaryotic translation initiation factor 4, gamma 1 | 7.8548 |
| NM_007956 | Esr1 | Estrogen receptor 1 (alpha) | -1.0539 |
| NM_010175 | Fadd | Fas (TNFRSF6)-associated via death domain | -1.0539 |
| NM_007987 | Fas | Fas (TNF receptor superfamily member 6) | 4.4745 |
| NM_008064 | Gaa | Glucosidase, alpha, acid | 2.9001 |
| NM_019749 | Gabarap | Gamma-aminobutyric acid receptor associated protein | -1.3453 |
| NM_020590 | Gabarapl1 | Gamma-aminobutyric acid (GABA) A receptor-associated protein-like 1 | 2.4893 |
| NM_026693 | Gabarapl2 | Gamma-aminobutyric acid (GABA) A receptor-associated protein-like 2 | 2.6761 |
| NM_008228 | Hdac1 | Histone deacetylase 1 | -1.0539 |
| NM_008244 | Hgs | HGF-regulated tyrosine kinase substrate | 8.7781 |
| NM_010480 | Hsp90aa1 | Heat shock protein 90, alpha (cytosolic), class A member 1 | 1.2786 |
| NM_031165 | Hspa8 | Heat shock protein 8 | 13.819 |
| NM_010414 | Htt | Huntingtin | -1.0539 |
| NM_010503 | Ifna2 | Interferon alpha 2 | -1.0539 |
| NM_010504 | Ifna4 | Interferon alpha 4 | -1.0539 |
| NM_008337 | Ifng | Interferon gamma | -1.0539 |
| NM_010512 | Igf1 | Insulin-like growth factor 1 | 2.0025 |
| NM_008387 | Ins2 | Insulin II | -1.0539 |
| NM_008326 | Irgm1 | Immunity-related GTPase family M member 1 | 5.6916 |
| NM_025735 | Map1lc3a | Microtubule-associated protein 1 light chain 3 alpha | -1.0231 |
| NM_026160 | Map1lc3b | Microtubule-associated protein 1 light chain 3 beta | -1.0001 |
| NM_011951 | Mapk14 | Mitogen-activated protein kinase 14 | 20.1316 |
| NM_016700 | Mapk8 | Mitogen-activated protein kinase 8 | 1.5477 |
| NM_008689 | Nfkb1 | Nuclear factor of kappa light polypeptide gene enhancer in B-cells 1, p105 | 5.7645 |
| NM_181414 | Pik3c3 | Phosphoinositide-3-kinase, class 3 | 3.8136 |
| NM_020272 | Pik3cg | Phosphoinositide-3-kinase, catalytic, gamma polypeptide | 2.2252 |
| NM_001081309 | Pik3r4 | Phosphatidylinositol 3 kinase, regulatory subunit, polypeptide 4, p150 | 15.0985 |
| NM_001013367 | Prkaa1 | Protein kinase, AMP-activated, alpha 1 catalytic subunit | 12.3193 |
| NM_178143 | Prkaa2 | Protein kinase, AMP-activated, alpha 2 catalytic subunit | -1.0539 |
| NM_008960 | Pten | Phosphatase and tensin homolog | 2.4644 |
| NM_009000 | Rab24 | RAB24, member RAS oncogene family | 3.138 |
| NM_009029 | Rb1 | Retinoblastoma 1 | 11.4974 |
| NM_026446 | Rgs19 | Regulator of G-protein signaling 19 | 8.6577 |
| NM_028259 | Rps6kb1 | Ribosomal protein S6 kinase, polypeptide 1 | 2.3843 |
| NM_009221 | Snca | Synuclein, alpha | -1.0539 |
| NM_011018 | Sqstm1 | Sequestosome 1 | 1.9956 |
| NM_011577 | Tgfb1 | Transforming growth factor, beta 1 | 4.032 |
| NM_009373 | Tgm2 | Transglutaminase 2, C polypeptide | 21.6817 |
| NM_145570 | Fam176a | Family with sequence similarity 176, member A | -1.0539 |
| NM_175502 | Tmem74 | Transmembrane protein 74 | -1.0539 |
| NM_026013 | Dram2 | VDNA-damage regulated autophagy modulator 2 | 1.642 |
| NM_013693 | Tnf | Tumor necrosis factor | 410.9451 |
| NM_009425 | Tnfsf10 | Tumor necrosis factor (ligand) superfamily, member 10 | -1.0539 |
| NM_011640 | Trp53 | Transformation related protein 53 | 4.7512 |
| NM_011642 | Trp73 | Transformation related protein 73 | -1.0539 |
| NM_009469 | Ulk1 | Unc-51 like kinase 1 (C. elegans) | 1.7508 |
| NM_013881 | Ulk2 | Unc-51 like kinase 2 (C. elegans) | 3.1034 |
| NM_178635 | Uvrag | UV radiation resistance associated gene | 12.2997 |
